# Structural-profiling of low molecular weight RNAs by nanopore trapping/translocation using *Mycobacterium smegmatis* porin A

**DOI:** 10.1038/s41467-021-23764-y

**Published:** 2021-06-07

**Authors:** Yuqin Wang, Xiaoyu Guan, Shanyu Zhang, Yao Liu, Sha Wang, Pingping Fan, Xiaoyu Du, Shuanghong Yan, Panke Zhang, Hong-Yuan Chen, Wenfei Li, Daoqiang Zhang, Shuo Huang

**Affiliations:** 1 State Key Laboratory of Analytical Chemistry for Life Sciences, School of Chemistry and Chemical Engineering, Nanjing University, Nanjing, China; 2 Chemistry and Biomedicine Innovation Center (ChemBIC), Nanjing University, Nanjing, China; 3 College of Computer Science and Technology, Nanjing University of Aeronautics and Astronautics, MIIT Key Laboratory of Pattern Analysis and Machine Intelligence, Nanjing, China; 4 Collaborative Innovation Center of Advanced Microstructures, National Laboratory of Solid State Microstructure, Department of Physics, Nanjing University, Nanjing, China

**Keywords:** Single-molecule biophysics, Nanobiotechnology, Characterization and analytical techniques

## Abstract

Folding of RNA can produce elaborate tertiary structures, corresponding to their diverse roles in the regulation of biological activities. Direct observation of RNA structures at high resolution in their native form however remains a challenge. The large vestibule and the narrow constriction of a *Mycobacterium smegmatis* porin A (MspA) suggests a sensing mode called nanopore trapping/translocation, which clearly distinguishes between microRNA, small interfering RNA (siRNA), transfer RNA (tRNA) and 5 S ribosomal RNA (rRNA). To further profit from the acquired event characteristics, a custom machine learning algorithm is developed. Events from measurements with a mixture of RNA analytes can be automatically classified, reporting a general accuracy of ~93.4%. tRNAs, which possess a unique tertiary structure, report a highly distinguishable sensing feature, different from all other RNA types tested in this study. With this strategy, tRNAs from different sources are measured and a high structural conservation across different species is observed in single molecule.

## Abstract (web summary)

Nanopores have been used for direct observation of RNA structure in native environments but have limited RNA differentiation capabilities. Here, the authors report on the use of Mycobacterium smegmatis porin A nanopores for the trapping and translocation identification of microRNA, siRNA, tRNA and ribosomal RNA.

## Introduction

The functional diversity of RNA stems in part from its ability to fold into elaborate tertiary structures that can specifically bind with ligands to regulate cellular activities^1,2^. Many unknown biological roles of RNA have been discovered^3,4^, leading to a growing demand for determination of RNA tertiary structures. Classical structural biology techniques including X-ray crystallography^5^ and NMR spectroscopy^6,7^ have contributed most to the RNA tertiary structure determination, preferentially those with small RNA architectures^8^. As a complement, cryo-electron microscopy (cryoEM) plays an increasingly important role in unveiling the structures of larger (>50 kDa) RNA molecules^9,10^. Emerging techniques such as single-molecule Förster Resonance Energy Transfer (smFRET)^11^ and single-molecule force spectroscopy^12^ have also been applied to probe RNA structure and interaction dynamics at the molecular level. However, high end equipment and laborious efforts in sample preparation are required and the risk of perturbing non-covalent interactions within the RNA structure is also present. As a consequence, direct interrogation of tertiary structures of RNA in its native state remains a challenge.

RNA structures can be probed by solid state nanopores^13–17^ and clinical applications such as the quantification of severe acute respiratory syndrome coronavirus 2 were as well demonstrated^14^. However, the thickness of a solid state nanopore prohibits it from producing refined sensing information, limiting its resolution to clearly resolve structurally similar RNA structures. Besides, the geometric reproducibility of a solid state nanopore remains a technical bottleneck, reducing the consistency of sensing when different batches of pores are used. Biological nanopores represent a growing family of channel proteins used for single-molecule sensing^18^. Emerging nanopores such as ferric hydroxamate uptake component A (FhuA) or aerolysin are capable of performing sensing of nucleic acids^19^, protein–protein interactions^20^ or amino acids^21^ with a high accuracy and consistency. Previous studies of transfer RNA (tRNA) using biological nanopores were carried out with wild type α-hemolysin (α-HL)^22^. However, chemical ligation with a leading strand is required and the acquired information reflects the difference of unfolding kinetics or the primary sequence rather than the overall tertiary structures of different tRNAs, largely due to a limited size of the pore constriction. To permit passage of large biomolecules, recent efforts have been made to develop biological nanopores with large constrictions. These pores include Cytolysin A (ClyA)^23^, Phi29 connector protein^24^, Fragaceatoxin C (FraC)^25^, FhuA^20^, and Pleurotolysin A (PlyA)/Pleurotolysin B(PlyB)^26^, with which dsDNA, proteins, or protein-small molecule complexes were thoroughly investigated. However, to the best of our knowledge studies of such complexes with RNA tertiary structures have not yet been carried out. These large pores are also associated with various issues such as short storage time^23^, non-uniform pore assembly^27^, or spontaneous gating when a large potential is applied^27^.

*Mycobacterium smegmatis* porin A (MspA) is a conically shaped biological nanopore composed of rigid β-barrel structures^28^. Previous reports indicate that the pore in an octameric form possesses an incredible stability and consistency against extreme conditions^29^. Its narrow constriction, measuring ~1.2 nm in diameter is advantageous in applications of nanopore sequencing^30^ or nanopore force spectroscopy^31^. On the other side, its large vestibule, which measures ~4.8 nm in diameter, would permit transient accommodation of a large analyte in its native form by nanopore trapping. Surprisingly, this geometric advantage has however been ignored since its original report.

We here propose a sensing mode with MspA, termed nanopore trapping/translocation, with which direct discrimination between differently structured low molecular weight (LMW) RNAs such as miRNA, overhanged siRNA, blunt siRNA, tRNA, or 5 S rRNA is reported. The RNA structure is profiled in its folded form during trapping. Translocation is not strictly needed and no denaturant or sample ligation is required. Complementary to existing developments of large channel proteins, advantages such as the efficiency of pore preparation, the ease of spontaneous pore insertion, the high consistency of pore assembly, the long storage time, and a high spatial resolution are all gained (Supplementary Figs. 1 and 2).

## Results

### Single-molecule sensing of miRNA

**Fig. 1:**
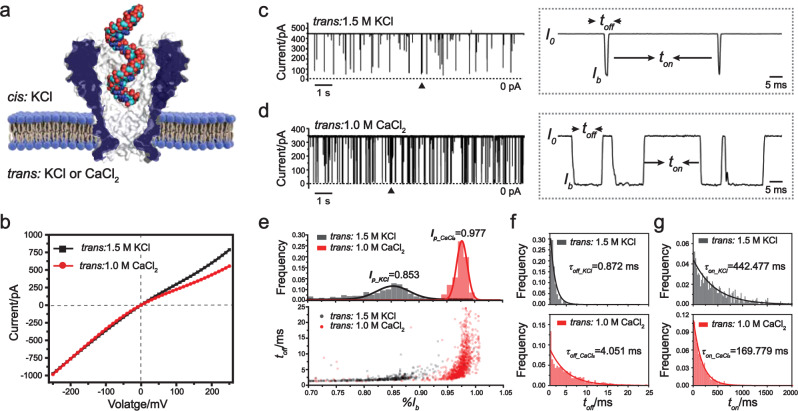
Retarded translocation of hsa-miR-21 through MspA. **a** A schematic diagram of hsa-miR-21 translocation through MspA. A single MspA is inserted in a lipid membrane separating the *cis* and the *trans* chambers. The *cis* chamber was filled with 1.5 M KCl buffer and the *trans* chamber with a 1.5 M KCl or a 1 M CaCl_2_ buffer. Hsa-miR-21 was added to *cis* with a final concentration of 200 nM. A transmembrane potential of +150 mV was continuously applied. **b** Current–voltage (*I*–*V*) curves of MspA in the presence of 1.5 M KCl (black) or 1 M CaCl_2_ (red) in *trans*. Different combinations of electrolyte buffers were applied and no analytes were added. **c** A representative trace containing successive hsa-miR-21 translocations. The measurement was performed with a 1.5 M KCl buffer in both *cis* and *trans*. Dashed box: a zoomed-in view of the section marked with a triangle on the trace. The open pore current (${I}_{o}$), blockage current (${I}_{b}$), dwell time (${t}_{{off}}$) and inter-event duration (${t}_{{on}}$) are marked. **d** A representative trace containing successive hsa-miR-21 translocations. The measurement was performed with a 1.5 M KCl buffer in *cis* and a 1 M CaCl_2_ buffer in *trans*. Dashed box: a zoomed-in view of the section marked with a triangle on the trace. In this condition, translocation events appear more frequently and are systematically retarded when **c**ompared with those in **c**. **e** Scatter plot of $\% {I}_{b}$ versus ${t}_{{off}}$ for hsa-miR-21 translocations and corresponding histogram of $\% {I}_{b}$. $\% {I}_{b}$ is defined as $({{I}_{o}-I}_{b})/{I}_{o}$. $\% {I}_{b}$ is larger and more uniformly distributed when a 1 M CaCl_2_ buffer in *trans* was applied (red). **f** The event histogram of ${t}_{{off}}$ for hsa-miR-21 translocations. **g** The event histogram of ${t}_{{on}}$ for hsa-miR-21 translocations. The histogram in **f** and **g** was single exponential fitted according to the equation $y=a\ast\exp(-x/\tau)$. The mean dwell time (${\tau }_{{off}}$) or the mean inter-event interval (${\tau }_{{on}}$) was respectively derived from the fitting results. Events with a dwell time <1 ms were ignored during the statistics.

Electrophysiology measurements were performed as described in Methods using the M2 MspA mutant (D93N/D91N/D90N/D118R/D134R/E139K)^32^ (Fig. 1a). If not otherwise stated, this mutant is referred to as MspA throughout this paper. Following the recently developed nanopore sensing strategy^33^, in which the presence of a calcium flux around the pore vicinity extends the dwell time of nucleic acid translocation, a 1.5 M KCl buffer (1.5 M KCl, 10 mM HEPES, pH 7.0) was placed in *cis* and a 1 M CaCl_2_ buffer (1 M CaCl_2_, 10 mM HEPES, pH 7.0) was placed in *trans*. According to the current–voltage characterization, the placement of a 1 M CaCl_2_ buffer instead of a 1.5 M KCl buffer in *trans* reduces the open pore current only slightly when a positive potential is applied (Fig. 1b).

Hsa-miR-21, which is one of the first identified mammalian microRNAs (miRNA) and has been well investigated as multiple cancer biomarkers^34^, was custom synthesized and treated as a model miRNA to test the method (Supplementary Table 1). Experimentally, after the addition of hsa-miR-21 with a 200 nM final concentration to *cis* and with a + 150 mV constantly applied potential, successive resistive pulses immediately appeared in both experiments. The open-pore current (${I}_{o}$), the blockage amplitude (${I}_{b}$), the dwell time (${t}_{{off}}$), and the inter-event interval (${t}_{{on}}$) are defined in Fig. 1c, d. The percentage blockade $\% {I}_{b}$ is determined from $\left({{I}_{o}-I}_{b}\right)/{I}_{o}.$ With the buffer combination of 1.5 M KCl (*cis*)/1.5 M KCl (*trans*), translocation events of hsa-miR-21 appeared as quite short-residing spikes as demonstrated in Fig. 1c. However, with the combination of 1.5 M KCl (*cis*)/1 M CaCl_2_ (*trans*) while keeping all other conditions identical, the rate of event appearance was significantly increased. The event dwell time was dramatically extended and the blockage amplitude (${I}_{b}$) became more uniformly distributed (Fig. 1d). This difference is more quantitatively demonstrated in the event scatter plot of $\% {I}_{b}$ vs ${t}_{{off}}$ and the corresponding histogram of $\% {I}_{b}$, from which the mean blockage amplitude ${I}_{p}$ was determined from the Gaussian fitting results (Fig. 1e, Supplementary Table 2). Histograms of ${t}_{{off}}$ and ${t}_{{on}}$ for both conditions were demonstrated in Fig. 1f, g. The histograms were singly exponentially fitted, from the results of which the mean dwell time (${\tau }_{{off}}$) and the mean inter-event interval (${\tau }_{{on}}$) were derived. The results shown in Fig. 1c–g clearly demonstrate that with all other conditions identical, a change of electrolyte buffer in *trans* to CaCl_2_ resulted in a dramatic increase in the rate of event appearance and event dwell time. The higher rate of event appearance should result from an increased electroosmotic flow induced by coordination interactions between Ca^2+^ and amino acid residues in the pore lumen (Supplementary Fig. 3). Ca^2+^ is known to stabilize RNA structure via efficient electrostatic charge screening or coordination binding, which have contributed to the extended dwell time. We have also performed hsa-miR-21 sensing with other electrolyte buffer combinations (Supplementary Fig. 4) and different MspA mutants (Supplementary Fig. 5). These results further confirm that an asymmetric buffer combination and the choice of M2 MspA are optimal for RNA structural profiling.

### Single-molecule sensing of siRNA

**Fig. 2:**
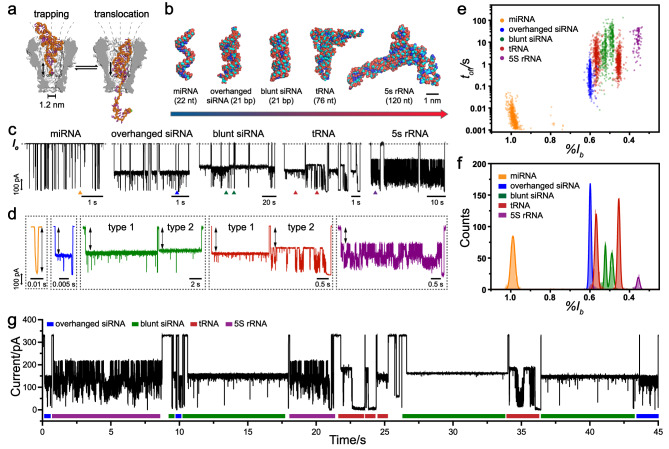
Distinguishing between LMW RNA tertiary structures with MspA. **a** The scheme of nanopore trapping/translocation using MspA. Current fluctuations between trapping and translocation reveal the RNA identity. A tRNA was employed as an example. **b** Representative RNA molecules studied in this manuscript. Five types of RNAs, including miRNA (single stranded, 22 nt), overhanged siRNA (double-stranded, 21 bp), blunt siRNA (double-stranded, 21 bp), tRNA (L shaped, 76 nt), and 5 S rRNA (Y shaped, 120 nt) were investigated. The measurements were carried out as described in Methods. MiRNA (has-miR-21), overhanged siRNA (SiFoxA1), blunt siRNA (luciferase siRNA), or tRNA (tRNA^phe^) were added to *cis* with a final concentration of 200 nM for each analyte. *E.coli* 5 S rRNA was added to *cis* with a final concentration of 10 nM. **c** Representative traces of successive translocations of miRNA (orange), overhanged siRNA (blue), blunt siRNA (green), tRNA (red), or 5 S rRNA (purple). The open pore current (${I}_{o}$) is marked with dashed lines. **d** Zoom-in views of representative translocation events from marked triangles of corresponding traces. Translocations of different RNA types result in highly distinguishable events features. MiRNA gives rise to fast spiky events. Overhanged siRNA produces two-step events. Blunt siRNA and tRNA both generate two types of events, termed type 1 and type 2. 5 S rRNA gives rise to three types of signals, which the most characteristic type is shown in the figure. The $\% {I}_{b}$ refers to the first-level blockade amplitude which is defined as marked in **d**. **e** A scatter plot of $\% {I}_{b}$ versus ${t}_{{off}}$ for five RNA samples. Events from five types of RNAs are clearly distinguishable. **f** The corresponding event histogram of $\% {I}_{b}$ of different RNA types. Black lines are Gaussian fittings to the data. **g** A representative trace during simultaneous sensing of siRNA, tRNA, and 5 S rRNA. Different RNA types (overhanged siRNA: 25 nM; blunt siRNA: 10 nM; tRNA: 400 nM; 5 S rRNA: 30 nM) were simultaneously added to *cis* side. Characteristic events from different RNA types are clearly recognized from the trace, which are marked with blue, green red, or purple bars respectively.

Small interfering RNA (siRNA), measuring 20–25 bp in length, appears as a RNA duplex with 2-nt 3′-overhangs or blunt ends and plays a central role in gene silencing^35^. This duplex of siRNA is conformationally more confined than that of dsDNA and is primarily in the A form^36^. The duplex of siRNA has a cross-sectional diameter of ~2.4 nm^37^, larger than that of the MspA constriction, indicating that a direct translocation of siRNA through MspA is geometrically restricted (Fig. 2a). To the best of our knowledge, previous attempts of siRNA translocation through an MspA have not been reported.

siFoxA1, which inhibits the expression of Forkhead protein FoxA1, is a 19-bp siRNA duplex with overhanging nucleotides on each end^38^ (Fig. 2b, Supplementary Table 1, Fig. 2b). After the addition of siFoxA1 to *cis* with a final concentration of 200 nM, the successive appearance of two-step blockade events was immediately observed during nanopore measurements (Supplementary Fig. 6). The first blockage level, measuring 0.600 ± 0.006 (*n* = 3) in $\bar{{I}_{p}}$ and a mean dwell time of a few hundred milliseconds, may represent the state when the siFoxA1 was accommodated in the pore vestibule. Immediately subsequent to this, the second blockage level, measuring 0.932 ± 0.004 (*n* = 3) in $\bar{{I}_{p}}$ and with a much shorter dwell time of a few milliseconds, may represent the state when the siFoxA1 was electrophoretically unfolded, allowing for a linearized, single-stranded portion of the analyte reaching the pore constriction and eventually generating a full translocation (Fig. 2c, d, Supplementary Fig. 6). By raising the applied potential to +200 mV, the dwell time of level 1 was significantly shortened (Supplementary Fig. 7). This is expected because an enhanced electrophoretic force would reduce the dwell time of siFoxA1 in its native folded state, further supporting the suggested model of translocation. Though the siRNA eventually translocates through the pore, the most characteristic event feature $\bar{{I}_{p}},$ measuring 0.600 ± 0.006 (*n* = 3) was obtained during the trapping stage.

Luciferase siRNA^39^, a 21-bp duplex and an inefficient silencing structure^40^, was employed as a model blunt siRNA (Fig. 2b, Supplementary Table 1). With the addition of luciferase siRNA to *cis* with a final concentration of 200 nM, two types of event, termed type 1 and type 2, were immediately observed (Fig. 2c, d, Supplementary Fig. 8), which are clearly distinguished from those produced by siFoxA1. Specifically, the type 1 event demonstrates a mean blockage amplitude ($\bar{{I}_{p}}$) of 0.490 ± 0.010 (*n* = 3, Supplementary Table 3). The type 2 event demonstrates a blockade with a mean blockage amplitude ($\bar{{I}_{p}}$) of 0.533 ± 0.004 (*n* = 3, Supplementary Table 3). Because the blunt ends are hard to be unzipped to reach the pore constriction (Supplementary Fig. 9), the events generally demonstrate shallower, longer residing, and less noisy a blockage level than those generated by siFoxA1 (Supplementary Fig. 8). The two types of events may thus result from blunt siRNA trapped by MspA in an opposite direction. Considering that the overall length and structure are similar, this comparison demonstrates that nanopore trapping/translocation by MspA can efficiently resolve minor structural differences between RNAs.

### Single-molecule sensing of tRNA

Transfer RNA (tRNA) is another intensively studied and well-known model in RNA structural biology. Its secondary structure is composed of four domains: the acceptor stem, the D-arm, the T-arm, and the anticodon loop (Supplementary Fig. 10). In three-dimensional space, these domains fold into an L-shaped tertiary structure, in which the anticodon loop and the acceptor stem respectively form the two ends of the L-shaped geometry (Supplementary Fig. 10). Judging from a visual inspection of its tertiary structure, tRNA, in its native form cannot directly translocate through MspA. However, it nevertheless fits into the pore vestibule and may have multiple orientations when entering the pore, suggesting that it might generate a set of translocation characteristics when probed by MspA (Fig. 2a).

Purification of a specific type of tRNA is difficult due to the biochemical similarity of different types of tRNAs^41^. Reported tRNA isolation is quite labor intensive, involving ionic exchange chromatography, solvent extraction, countercurrent extraction, chromatography on benzyl-DEAE-cellulose, and reverse-phase chromatography^41^. However, phenylalanine specific tRNA, abbreviated here as tRNA^phe^, is unique because it can be simply obtained with high purity by elution from a benzylated DEAE-cellulose column with a gradient of NaCl^42^. Brewer’s yeast tRNA^phe^, which was extracted as described above^42^, is commercially provided by Sigma-Aldrich and was employed as a representative tRNA in follow-up studies.

During a nanopore measurement (Methods), Brewer’s yeast tRNA^phe^ was added to *cis* with a final concentration of 200 nM. Successive long residing and fluctuating translocation events were subsequently observed, among which two types of events, tentatively termed tRNA type 1 or type 2 events, demonstrate a high reproducibility in their event characteristics (Fig. 2c, d, Supplementary Fig. 10). When the measurements were carried out with 1.5 M KCl (*cis*)/1.5 M KCl (*trans*), tRNA^phe^ translocation results in events with non-uniform characteristics. Previously observed type 1 and type 2 events have completely disappeared (Supplementary Fig. 11). This suggests that the presence of the calcium flux may have helped to stabilize tRNA tertiary structures during nanopore sensing (Supplementary Fig. 11)^43^. Specifically, the tRNA type 1 event demonstrates a single-step blockade with a mean blockage amplitude $\bar{{I}_{p}}\,$ of 0.567 ± 0.004 (*n* = 3, Supplementary Table 3). The tRNA type 2 event contains a well-defined upper blockage level (level 1) with an $\bar{{I}_{p}}$ of 0.453 ± 0.002 (*n* = 3, Supplementary Table 3). Besides, the event contains persistent transitions to deeper blockage levels and eventually ends with a quite deep pore blockage (level 2) measuring 0.997 ± 0.010 in $\bar{{I}_{p}}\,$ (*n* = 3, Supplementary Table 3) before being restored to the open pore level. The shallow blockage amplitude (${I}_{p}$) in type 1 or level 1 of type 2 suggests that the tRNA was in the form of partial translocation, leaving a large remaining space in the pore vestibule unoccupied and resulting in a large residual current. The highly distinguishable differences in ${I}_{p}$ between these two types of events may result from two distinct tRNA trapping orientations. According to its tertiary structure, either the anticodon loop or the acceptor stem of tRNA may face the pore constriction during translocation.

To further explore this phenomenon, nanopore measurements with tRNA^phe^ were carried out with applied voltages varying between +125 and +225 mV. Both tRNA type 1 and type 2 events were still observed. In general, the residence times of all type 1 events were systematically extended when the applied voltage was increased (Supplementary Fig. 12, Supplementary Table 4), indicating that a type 1 event actually represents trapping of the tRNA without an eventual passage through the pore. In this case, a higher electrophoretic force would keep the trapped tRNA more tightly in the pore vestibule before escaping back to the *cis* chamber, resulting in a systematically extended dwell time for the event. Without any observation of further pore blockages in any type 1 event, a full translocation with this orientation seems to be impossible. This suggests that the anticodon loop of the tRNA tertiary structure, which forms a covalently closed molecular circle, is facing the pore constriction during translocation (Supplementary Fig. 10). The overall dwell time of type 2 events however behaves in the opposite sense (Supplementary Fig. 12, Supplementary Table 4), indicating that the type 2 event actually represents a kind of translocation during which the tRNA was unfolded, leading eventually to a full translocation. This hypothesis is reinforced by the observation of persistent attempts of the tRNA to reach a further pore blockage level, as observed from the fluctuations below the level 1 blockage state. The acceptor stem, which has a phosphorylated 5′ end and an overhanging 3′ end which contains a CAA tail for amino acid attachments, may facilitate electrophoretically driven unfolding of the tRNA structure, when facing the pore constriction (Supplementary Fig. 10). These findings have reinforced the speculation that two tRNA^phe^ translocation orientations were observed. The spatially asymmetric tRNA results in distinguishing of tRNA^phe^ translocation orientations, generating two tracks of sensing information for tRNA structural profiling.

### Single-channel recording of 5 S rRNA

5 S ribosomal RNA (5 S rRNA) is an integral component of the ribosome. Its small size (approximately 120 nt), conserved structure, and association with ribosomal proteins made it an ideal model RNA for studies of RNA structure^44^ and RNA–protein interactions^45^. The secondary structure of 5 S rRNA is composed of five helices (denoted I–V in roman numerals), four loops (B–E), and one hinge (A), which form a Y-shaped tertiary structure^46^. The loop C, loop E, and helix I are located at the three ends of the “Y” shape^46^. The structure shows a higher complexity than that of tRNA and might generate different event characteristics when probed by MspA.

5 S rRNA extracted from *E. coli* (Fig. 2b, Supplementary Fig. 13) was employed as a model analyte, which was added to *cis* with a final concentration of 10 nM. Three types of characteristic events were observed which might be corresponding to the three terminals of 5 S rRNA entering the pore, respectively (Supplementary Fig. 14). Specifically, the type 1 event appears as current oscillations below a characteristic blockade level with a mean blockage amplitude ($\bar{{I}_{p}}$) of 0.356 ± 0.003 (*n* = 3, Supplementary Table 3). The type 2 event starts with random current fluctuations. Then it becomes a single-step blockade (level 1, $\bar{{I}_{p}}\,$= 0.566 ± 0.017, *n* = 3) with many negative going spikes. The type 3 event demonstrates a two-step blockade and the mean blockage amplitude ($\bar{{I}_{p}}$) of the first step is 0.737 ± 0.005 (*n* = 3, Supplementary Table 3). Different event types were well distinguished from each other based on the results of their all-point histograms (Supplementary Fig. 15). Among the three types of events, the type 1 event demonstrates the most unique event shape and the highest appearance probability, which was considered the most characteristic event type of 5 S rRNA (Supplementary Fig. 16). By performing a voltage dependence assay, it was discovered that the type 1 event is a combination of trapping and translocation. A higher applied voltage would eventually drive the 5 S rRNA structure to unfold and translocate through the pore (Supplementary Fig. 17). Thus, the type 1 event is most likely to be the result of the helix I-down pose instead of any loop-down poses. The type 2 events, which never demonstrate any sign of successful translocation through the pore, should result from trapping of the structure with a loop-down pose (Supplementary Fig. 14). Whereas, the type 3 events, which are relatively short residing and much less frequent in appearance, always appear as translocation through the pore (Supplementary Fig. 14). Thus, the type 3 events should result from translocation of unfolded or fragmented 5 S rRNA considering that the large size of 5 S rRNA won’t easily permit its translocation through the pore. Rich sensing information generated by MspA trapping/translocation has provided a clear reference in recognition of 5 S rRNA in single-molecule. However, structural profiling of 5 S rRNA by nanopore has not yet been previously reported, to the best of our knowledge.

### Single-molecule RNA structural profiling

Hsa-miR-21, siFoxA1, luciferase siRNA, tRNA^phe^, and 5 S rRNA demonstrate an increased complexity in their overall structures. These differences in the structure were all discriminable by the same pore MspA, utilizing the large opening of the pore vestibule and an overall conical pore geometry (Fig. 2a, b and Supplementary Fig. 18). The event scatter plots of $\% {I}_{b}$ vs ${t}_{{off}}$ of different RNA types are shown in Fig. 2e (Supplementary Table 3). For 5 S rRNA, the type 1 event, which is the most representative event type of 5 S rRNA, is demonstrated. For multi-step blockade events, the blockade amplitude of the first step was counted. Event characteristics generated by different analyte form highly distinguishable populations of distribution in the scatter plot. A corresponding event amplitude histogram is also demonstrated in Fig. 2f, in which 5 S rRNA results in the shallowest blockade, followed by tRNA^phe^, luciferase siRNA, siFoxA1, and hsa-miR-21. This is expected as RNAs with a larger tertiary structure have more difficulty accessing the pore constriction.

Simultaneous sensing of siFoxA1, luciferase siRNA, tRNA^phe^, and 5 S rRNA using MspA were also demonstrated (Fig. 2g). Different RNA types can be clearly recognized based on their distinct blockade characteristics. These results indicate that MspA, which has a conical shape, effectively distinguishes between a wide variety of RNA types for structural profiling. Although not demonstrated, other classical RNA structures, including kissing loop^47^, three-way junction^48^, pseudoknot^49^, kink-turn^50^, and G-quadruplex^51^ are in principle detectable by the same strategy and distinct event features are expected. Subsequent feature extraction and analysis can be labor-intensive or may be biased by human supervision. Events resulted from RNA structures with a higher order of complexity may also require multiple parameters in the description of their characteristics. A highly intelligent and user-friendly computer algorithm is urgently needed to cope with these challenges.

### Machine learning assisted RNA identification

Machine learning is a branch of artificial intelligence research, whose aim is to build computerized algorithms which learn from input data without focusing on programming. This concept demonstrates a generality suitable for analyzing nanopore sensing data, as previously reported^13,52–55^. Event characteristics of siRNA, tRNA, and 5 S rRNA demonstrate a high consistency when probed by MspA, and such data are well suited for the construction of a machine learning algorithm aiming to automatically recognize different RNA structures. To begin with, raw time traces containing nanopore sensing events were first automatically segmented to generate discrete nanopore events (Supplementary Fig. 19). To form model training sets, model events including 118 overhanged siRNA (siFoxA1) events, 176 blunt siRNA (luciferase siRNA) type 1 events, 161 blunt siRNA (luciferase siRNA) type 2 events, 143 tRNA (tRNA^phe^) type 1 events, 155 tRNA (tRNA^phe^) type 2 events, 133 5 S rRNA (*E.coli* 5 S rRNA) events and 134 “others” events were used. All these training events have known identities since they were generated during measurements involving a sole, known analyte. Here, events defined as “others” were abnormal nanopore events mainly caused by nanopore clogging or spontaneous gating (Supplementary Fig. 20). These events were also included in the training dataset serving as interfering events, reinforcing the robustness of the training. The type 1 or type 2 events were separately labeled according to their highly discriminable $\% {I}_{b}$ values (Fig. 2c, Supplementary Table 3).

**Fig. 3:**
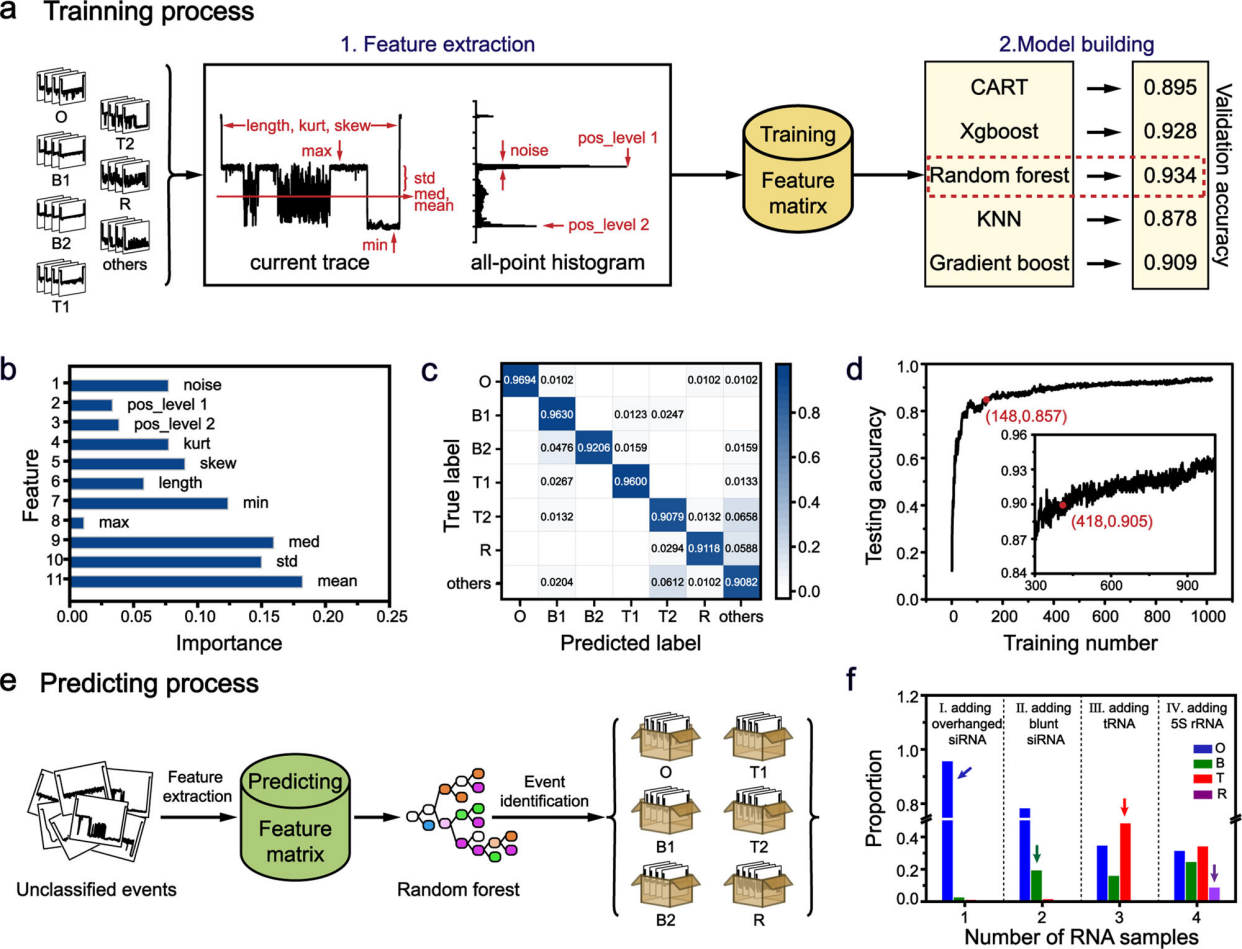
Machine learning assisted RNA type identification. **a** The flow diagram of the training process. Seven classes of events, including overhanged siRNA (O), blunt siRNA type 1 (B1), blunt siRNA type 2 (B2), tRNA type 1 (T1), tRNA type 2 (T2), 5 S rRNA (R) and others were formed as the training dataset. Eleven features were extracted to form a feature matrix. The training dataset was then split to training subset and validation subset, of which the validation subset serves to verify the accuracy of the classifier model. The accuracy was defined as the ratio of events in the validation subset that were correctly identified. Five classifiers were investigated to identify the best performing model, for which the random forest model has demonstrated the highest accuracy, scoring 0.934. **b** Feature importance obtained from the Random forest model. All features play a role in RNA event recognition. **c** The confusion matrix of RNA classification. Accuracies of the testing set were obtained by the Random forest model. The testing set was composed of 98 overhanged siRNA events, 81 blunt siRNA type 1 events, 63 blunt siRNA type 2 events, 75 tRNA type 1 events, 76 tRNA type 2 events, 68 5 S rRNA, and 98 “others”. **d** The learning curve with varying sample size of the training set. When the sample of the training set exceeds 148, the accuracy of validation has reached 0.85. When it exceeds 418, the accuracy saturates at ~0.90. **e** The flow diagram of the predicting process. The raw current traces of mixed samples were segmented into separate, unclassified events. Event features were extracted and serve as predicting set, which was subsequently recognized and sorted using the Random forest model. **f** The proportion of different RNA events determined with the Random forest model. The arrows indicate the proportion of RNA newly added. Four sets of data were recorded when overhanged siRNA (blue), blunt siRNA (green), tRNA (red), and 5 S rRNA (purple) were sequentially added to *cis*.

The training process is composed of feature extraction and model building (Fig. 3a). During feature extraction, level 1 position (pos_level 1), level 2 position (pos_level 2), noise, dwell time (length), minimum (min), maximum (max), median (med), mean, standard deviation (std) kurtosis (kurt) and skewness (skew) of individual events were respectively extracted, forming a feature matrix for each event (Fig. 3a). The method of feature extraction is detailed in Supplementary Fig. 21. Then the training datasets were split into the training set for model training and the testing set for model testing. The training set was further randomly split by the 10-fold cross-validation into a training subset for model training and a validation subset for model parameter fine-tuning and model validation. The training process was performed 10 times during which the training dataset was randomly partitioned and performance bias is avoided. To build the model, five different classifiers, including Classification And Regression Tree (CART), Xgboost, Random Forest, KNN, and Gradient Boost were estimated. Due to a large variation of event length between event types, Deep-Learning was not selected for model building. Hyperparameters such as “n_setimators” from RandomFoest, “k value” from KNN were fine-tune by the validation subset. Each model accuracy score is computed by averaging the accuracy score of all model training. Among all five classifiers, the Random Forest model has scored the highest and became the optimum choice of model builder. The trained models were tested by the testing dataset. The phase of model testing outputs the classification accuracy, feature importance, confusion matrix and learning curve. The classification accuracy is computed by the quotient of correctly classified samples and total samples.

The feature importance was generated during model testing which demonstrates the relative importance of all nine features in event recognition (Fig. 3b). The confusion matrix results of model testing are demonstrated in Fig. 3c, from which the accuracy of overhanged siRNA, blunt siRNA type 1 and siRNA type 2, tRNA type 1 and type 2, 5 S rRNA are 0.9694, 0.9630,0.9206, 0.9600, 0.9079, and 0.9118, respectively. To estimate the efficiency of the model, the accuracy was estimated with a varying amount of input data during model testing to form a learning curve, which suggested that an overall judgment accuracy of 85% can be achieved with an input of only 148 training events, randomly selected from the whole training sets (Fig. 3d).

The model was employed to predict events with unknown identities (Fig. 3e). Nanopore measurements were carried out with sequential addition of overhanged siRNA, blunt siRNA, tRNA, and 5 S rRNA. A twenty-minute trace was recorded for each condition. The recorded data forms the predicting datasets, which were subsequently identified by the previously trained model (Supplementary Movie 1). As shown in the histogram of event recognition (Fig. 3f, Supplementary Fig. 22), an obvious rise in the proportion of the corresponding RNA event emerges after each addition. This efficiently assists automatic nanopore sensing of different RNA structures, and is especially advantageous in RNA identification from mixed samples.

### Molecular dynamics study of tRNA trapping/translocation

**Fig. 4:**
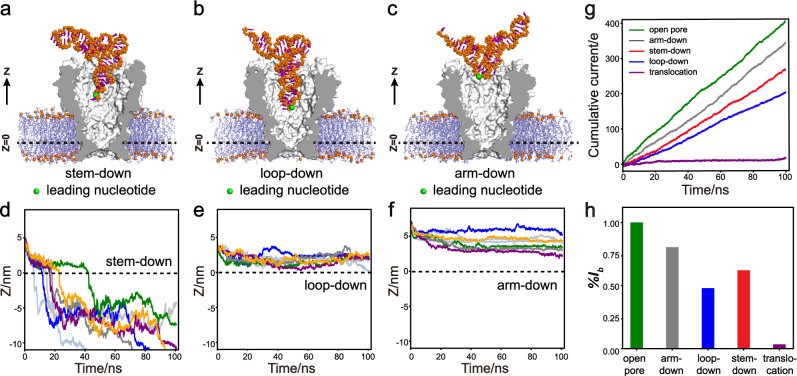
MD analysis of tRNA translocations. **a–c** Equilibrated structures of tRNA entering an MspA nanopore. The conformations are respectively termed as stem-down **a**, loop-down **b**, or arm-down **c**. The green sphere marked on each conformation referred to the leading nucleotide, which was used to characterize the position of tRNA in the following simulation results. **d–f** The z-coordinates of the leading nucleotide as a function of time for the simulations with the stem-down **d**, the loop-down **e**, and the arm-down **f** conformation. Seven trajectories were shown for each condition. The results show that the stem-down conformation has a higher tendency to translocate through the pore, while the other two conformations cannot reach the pore constriction to initiate a translocation. **g** Simulated cumulative ion currents through the pore at the open pore (green), the arm-down (gray), the stem-down (red), the loop-down (blue), and the tRNA translocation (purple) state. The external electric field was 0.09 V/10 nm, which corresponds to a voltage bias of ~+150 mV. The tRNA does not have obvious movement along the *z*-axis during the simulation timescale with such a low voltage. The slopes of the cumulative currents represent the ion current values. **h** The derived ionic currents for different states of sensing. All values were scaled so that the open pore current reports 1.

Among all tested analyte, tRNA demonstrates two highly characteristic types of events. Experimentally, these two event types respectively demonstrate trapping (type 1) and translocation (type 2) of tRNA when probed by MspA (Supplementary Fig. 10). Since the overall structure of tRNA is multi-branched, the origin of the two event types likely results from different orientations of tRNA entering the pore. To reveal how it determines the blockade amplitude and the kinetics of tRNA during nanopore sensing, all-atom MD simulations were performed (Methods). The simulations were initiated by placing a tRNA with different start orientations immediately above the pore vestibule without any direct contact with the pore. The conformations demonstrating these orientations, which were respectively referred to as the stem-down, the loop-down, or the arm-down orientation, were equilibrated and demonstrated in Fig. 4a–c. To further characterize the translocation process of tRNA, we probed the z-coordinate of the leading nucleotide (green sphere in Fig. 4a–c) during a 100-ns simulation. Here, Z = 0 corresponds to the narrowest region of MspA (Fig. 4a–c), which is the center of mass of the C_α_ atoms of the N90 in all eight subunits. Thus, a result of Z < 0 demonstrates that the leading nucleotide has successfully translocated through the pore. Experimentally, trapping/translocation of tRNA lasts ~seconds when probed by MspA, which is far beyond the accessible timescale of conventional MD simulations. In a previous work^56^, the whole vestibule of MspA was removed to speed up the calculation so that a ~μs timescale in a single trajectory of the all-atom simulations was achieved. However, the vestibule of MspA is critical to accommodate large RNA structures and a ~μs timescale is still much shorter than that took for nanopore trapping/translocation. Alternatively, to observe the full process of nanopore trapping/translocation within a feasible simulation timescale, a higher voltage was applied to speed up the process. However, the corresponding ionic current is derived by switching the applied voltage to +150 mV. To avoid the formation of electroporation, the positions of lipid molecules were restrained. The simulations were identically carried out for all three different orientations of tRNA entering the pore for a qualitative comparison.

Figure 4d–f shows representative trajectories from seven independent simulations when respectively simulated with three different conformations. The results show that tRNA with the stem-down conformation can translocate through the pore constriction much more easily than the others. In all simulations with the stem-down pose, the leading nucleotide has successfully translocated through the MspA porin within 100 ns. Whereas in the simulations with the other two tRNA poses, no successful translocation events were observed within the simulation timescale. Further simulations suggest that the successful translocation with the stem-down conformation is coupled with the unfolding of tRNA (Supplementary Fig. 23, Supplementary Movie 2). At the early stage of the simulation, tRNA involves dramatic deformation without disrupting the base-pair hydrogen bonds (H-bond) as indicated by the increase of the root mean square deviation (RMSD) and the relatively stable values of the H-bond (Supplementary Fig. 23). Due to the deformation, tRNA can reach a deeper position of MspA, which is followed by the tRNA unfolding and the successful translocation of the leading nucleotide through the pore constriction, as shown by the drop of the reaction coordinate Z, the decrease of the H-bond, and the increase of the RMSD (Supplementary Fig. 23). The translocation processes with the other two conformations are also provided in Supplementary Movie 3 and 4.

The different analyte-pore interactions caused by different conformations of tRNA lead to distinctive ionic currents. To quantitatively compare the resulting ionic current for the different conformational states of the system, the external electric field was switched to 0.09 V/10 nm, which corresponds to a voltage bias of ~+150 mV as used in the experiments. Following a previous study^57^, the instantaneous ionic current was calculated based on the coordinates of the ions. Since the instantaneous ionic current has large fluctuations, we first calculated the cumulative currents. Then the ionic currents were derived from the slope of the cumulative currents by linear fitting. In addition to the above-mentioned three simulation systems, we also performed ion current simulations for the systems without tRNA (open pore) and with the tRNA translocating through the pore (Z < 0). As shown in Fig. 4g, h, the simulations of the open pore state of MspA shows the highest ion current. After tRNA was trapped into the pore vestibule, the ionic currents abruptly decreased, leading to a current blockade event. Compared to the stem-down conformations, the loop-down reports a higher current blockage, consistent with the experiment observation that level 1 of type 2 event is always higher than that of type 1 event. The current almost vanishes when the tRNA is translocating through the pore constriction, which well describes the state of level 2 of a tRNA type 2 event. These results were similarly observed when the voltage was further up-regulated (Supplementary Fig. 24). To summarize, the above results by MD simulations have well explained the possible origin of two tRNA event types, especially the type 2 event which corresponds to tRNA translocation driven by voltage driven unfolding. The type 1 event, which is a trapping event (Supplementary Fig. 12), is likely resulted from the loop-down orientation instead of the arm-down orientation. The arm-down orientation demonstrates a shallow trapping depth from the simulation, which is less likely to happen than the loop-down orientation when experimentally measured. MD simulation was also similarly carried out for 5 S rRNA (Supplementary Fig. 25, Supplementary Movie 5), which has demonstrated details of molecular translocation of a much larger RNA structure. Voltage driven unfolding was also observed in the simulation initiated from a helix I down trapping orientation.

### Event feature conservation for tRNAs from different sources

Previous crystallographic studies indicate that with the exception of particular mammalian mitochondrial tRNAs, tRNAs of a widely divergent phylogenetic origin demonstrate a highly conserved L-shaped tertiary conformation^58^. With this knowledge in mind, the structure-induced nanopore events of brewer’s yeast tRNA^phe^ might be generally applied to a much wider variety of tRNAs from different sources. To explore this speculation, we performed nanopore sensing of the total tRNAs from brewer’s yeast and from *E.coli*, both supplied by Sigma-Aldrich.

Gel electrophoresis was performed for both tRNA samples, from which the yeast total tRNAs have the desired purity but the *E.coli* total tRNAs contain noticeable contaminations, including 5 S rRNA and other higher molecular weight RNAs^59^ (Supplementary Fig. 26). To avoid interference from contaminants, *E.coli* tRNA was purified by RNA recovery from a polyacrylamide gel prior to nanopore measurements (Supplementary Fig. 27).

**Fig. 5:**
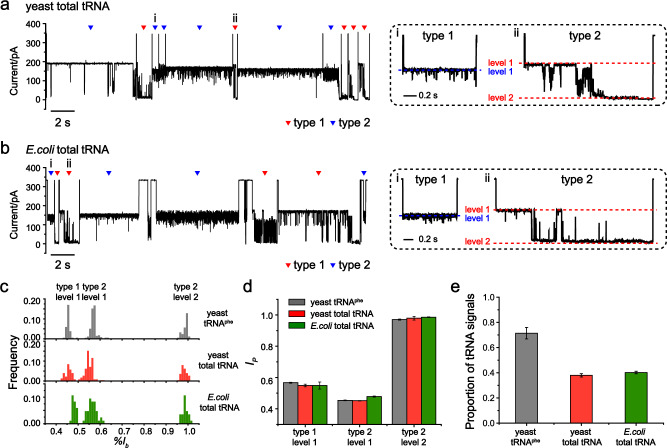
Single-molecule sensing of total tRNAs from different species. All measurements were performed as described in Methods. Yeast tRNA was added to *cis* with a final concentration of 20 ng/μL. *E.coli* tRNA was added to *cis* with a final concentration of 2 ng/μL. Trace segmentation and event recognition were performed with the custom machine learning algorithm (Fig. 3a). **a** A representative trace containing successive yeast tRNA translocation through MspA. Two types of events, termed type 1 (blue triangle) and type 2 (red triangle), were observed, forming the majority of all events that were recorded. Dashed box: Zoomed-in views of representative type 1 and type 2 events, which are respectively marked with i and ii on the trace. The type 1 event has a single blockade level (level 1). The type 2 event contains two blockade levels (level 1 and level 2). **b** A representative trace containing successive *E.coli* tRNA translocation through MspA. Two types of events, termed type 1 (blue triangle) and type 2(red triangle) events, were also observed, forming the majority of all events that were recorded. Dashed box: Zoomed-in views of representative type 1 and type 2 events, which are respectively, marked with i and ii on the trace. **c** The event histogram of blockade amplitude of type 1 and type 2 events (gray: tRNA^phe^. red: yeast total tRNA. green: *E.coli* total tRNA). Please note that the current fluctuations between level 1 and 2 show slight variations between events. This variation of fluctuation is more clearly observed in measurements with total tRNAs than those with tRNA^phe^ (Supplementary Fig. 10). However, the $\% {I}_{b}$ of level 1 and 2 of type 2 events are much more conserved. **d** Comparison of percentage blockage (${I}_{p}$) of type 1 and type 2 events acquired from different tRNA samples. **e** The proportion of characteristic tRNA events from different sources of tRNAs. Error bars in **d** and **e** represent standard deviation, *n* = 3 independent replicates.

During nanopore measurements (Methods), yeast tRNA or purified *E.coli* tRNA were respectively added to *cis* at a 20 ng/μl or 2 ng/μl final concentration. Representative traces were separately demonstrated in Fig. 5a, b. Characteristic tRNA type 1 and type 2 events, as previously defined when brewer’s yeast tRNA^phe^ was studied, were clearly observed from both traces. Figure 5c shows the event histogram of blockade characteristics of type 1 (level 1) and type 2 (level 1 and level 2) events induced by yeast tRNA^phe^, yeast tRNA, or *E.coli* tRNA. Generally, tRNA events from different sources or species demonstrate a high similarity in event statistics when probed by MspA. Statistical analysis from three independent experiments also showed that the ${I}_{p}$ of the three characteristic levels of yeast tRNA and *E.coli* tRNA translocation events is close to that from yeast tRNA^phe^ (Fig. 5d, Supplementary Table 5). The proportions of characteristic tRNA events from yeast tRNA and *E.coli* tRNA are also similar (Fig. 5e, Supplementary Table 6). These results reveal that tRNA characteristic events are highly conserved for tRNAs from different sources or species. Though the same conclusion has been previously drawn from crystallographic results^60–64^, this is the first demonstration of tRNA structural conservation from single-molecule observation, and acquired with natural samples in an aqueous buffer environment instead of samples in a static, crystallized form. In addition, the blockade current distributions of type 1 level 1 and type 2 level 2 appear slightly wider than that of yeast tRNA^phe^, possibly indicating that different tRNAs may show further distinguishable characteristics, though the general shape of event appears to be similar. The unique event characteristics along with the single-molecule resolution of the nanopore enables direct tRNA recognition from complex biological samples, such as a crude extract from the cell lysate in which a significant amount of interfering analyte is present.

### Direct tRNA identification from *E.coli* extracts

**Fig. 6:**
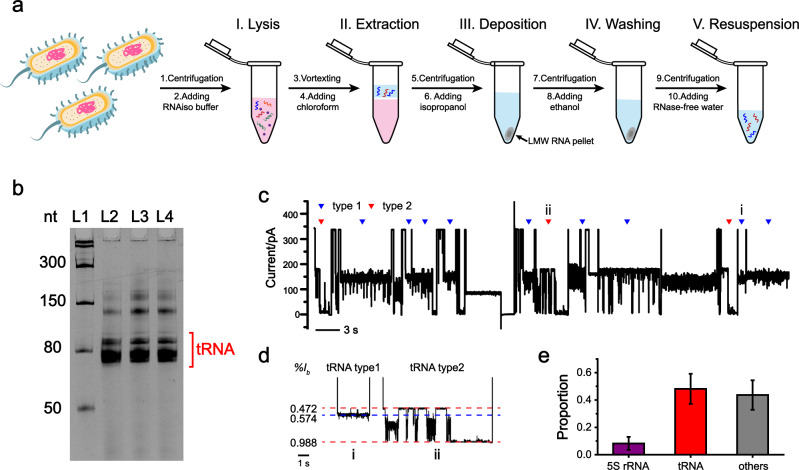
Direct identification of tRNA from *E.coli* LMW RNA extracts. **a** Isolation of LMW RNA from *E.coli*. (I) *E.coli* pellets were lysed in the RNAiso buffer (Takara). (II) LMW RNA was extracted with chloroform and retained in the supernatant. (III) After centrifugation, the supernatant was collected and added with isopropanol to precipitate all LMW RNA. (IV) The precipitant was collected and washed with 75% ethanol. (V) The LMW RNA was dissolved in ribonuclease (RNase)-free water. **b** Denaturing urea polyacrylamide gel electrophoresis (Urea-PAGE) analysis of *E.coli* LMW RNA, extracted as described in **a**. L1: low range RNA ladder. L2-L4: *E.coli* LMW RNA. The band corresponding to tRNA is marked on the gel. The uncropped gel is provided in Supplementary Fig. 28. **c** A representative trace containing successive translocation of *E.coli* LMW RNA through MspA. Characteristic tRNA type 1 and type 2 events are respectively marked with blue and red triangles. **d** Zoomed-in views of representative translocation events in **c**, which were marked with i and ii on the trace. **e** The proportion of tRNA translocation events (purple: 5 S rRNA. red: tRNA. gray: others). 48% of all acquired events were recognized as either tRNA type 1 or tRNA type 2 events. Error bars represent standard deviation, *n* = 3 independent replicates. The measurements in **c**–**e** were performed as described in Methods. *E.coli* LMW RNA was added to *cis* with a final concentration of 40 ng/μL. Trace segmentation and event recognition were performed with the custom machine learning algorithm (Fig. 3a).

To verify its feasibility, cultured *E.coli* (BL21) DE3 was lysed. All low molecular weight (LMW) RNA ( < 200 nt) was extracted by the small RNA extraction reagent from Takara, named RNAiso for Small RNA. The extraction procedure is schematically illustrated in Fig. 6a and detailed in Methods. The kit efficiently extracts all low molecular weight RNAs in the lysate, including tRNA, 5 S rRNA, miRNA, and siRNA^65^. Other than tRNA, the other RNAs may serve as interfering RNAs with which to test the robustness of the machine learning algorithm.

Prior to nanopore measurements, the extracted sample was first characterized by 12% denaturing urea polyacrylamide gel electrophoresis (Urea-PAGE) analysis (Fig. 6b, Supplementary Fig. 28). According to published reports^61^, the band with a molecular weight equivalent to ~80 nt corresponds to the tRNAs^65^. Nanopore sensing of LMW RNA was performed with 40 ng/μL LMW RNA in *cis*. A representative trace of a 70 s duration is shown in Fig. 6c. According to the custom machine learning algorithm, the characteristic type 1 and type 2 events were automatically identified, and are marked with triangles in Fig. 6c. Statistics show that the identified tRNA events have made up 48% of all detected translocation events (Fig. 6d, e, Supplementary Table 6). This is expected considering the possible interferences from 5 S rRNA, miRNA, or siRNA, simultaneously present in the lysate.

As a negative control, high molecular weight (HMW) RNAs (>200 nt) of *E.coli* (BL21) DE3 were extracted using MiniBEST Universal RNA Extraction Kit (Takara). This kit preferentially extracts all RNAs with a molecular weight >200 nucleotides (nt) according to the manufacturer’s protocol^66^. Detailed extraction procedures are described in Methods. Experimentally, from 1% agarose gel electrophoresis results, the sharp bands respectively correspond to the 23 S Ribosome RNA (rRNA) (2904 nt) and the 16 S rRNA (1542 nt) which is a good indication that the HMW RNA extraction was successful (Supplementary Fig. 29). 5 S rRNA (120 nt) and tRNA (70–90 nt) which cannot be efficiently extracted by the Takara kit, were not clearly visible in the gel.

Nanopore sensing of the HMW RNA extraction was performed with a 50 ng/μL final concentration of HMW RNA in *cis*. A representative 10 min trace is shown in Supplementary Fig. 30, from which long blockade events ranging from 1 to 60 s appear successively in the trace. These events may result from either 23 S rRNAs or 16 S rRNAs and show less defined event characteristics. However, they are clearly distinguishable from all tRNA events. Only 3.7% tRNA type 1 and no type 2 events were observed (Supplementary Fig. 30). Previous trials with tRNA containing samples all demonstrate both type 1 and type 2 events (Fig. 2c) of which type 2 is more characteristic in the identification of tRNA. In this case, without a simultaneous appearance of the tRNA type 2 event, the observation likely results from a minority of the events from HMW RNAs appearing similar to the tRNA type 1 event.

## Discussion

In summary, this paper presents a nanopore sensing strategy which directly distinguishes between RNA native structures utilizing the large vestibule of an MspA nanopore. Representative RNA analytes, including miRNA, siRNA, tRNA, or rRNA, generate rich sensing information during translocation which reports their identities unambiguously. We admit that RNA structural profiling by nanopore trapping/translocation may get complicated when structurally similar RNAs were simultaneously evaluated. However, compared with existing RNA detection methods based on hybridization^67,68^ or reverse transcription^69,70^, it requires no prior chemical treatment or amplification and a single molecule resolution is achieved. It thus serves as an alternative method for fast estimation of the expression level of a particular RNA, and is suitable for assessment of RNA integrity, stress-induced tRNA differential expression^71^ or tRNA cleavage derived fragments^72^. Acknowledging an overall rigidity and conical geometry of the pore, trapping by MspA also reports highly consistent and distinguishable event characteristics. To cope automatically and quantitatively with sensing events, a custom machine learning algorithm has been developed (Fig. 3a). Though machine learning has only been previously applied in few practices of nanopore sensing^13,53,54^, tools from artificial intelligence are gaining a growing importance in the field, in preparation of the era to be led by high throughput sensing^73^. With the above sensing strategy, tRNA which possesses an L-shaped tertiary structure, reports highly unique sensing characteristics. This unique feature also shows a high conservation between samples from different species (Fig. 5a) or sources (Fig. 5a).

Our results confirm that the vestibule of MspA can serve as a large constriction, complementary to the development of large pores such as ClyA^23^, Phi29 DNA connector^24^, FraC^25^, PlyA/PlyB^26^, or DNA nanopores^74^, however, the exceptional structural stability of MspA is advantageous for sample storage, long-term measurement and a low noise of measurement. Though not yet disclosed in this study, the strategy of nanopore trapping has as well been successfully used to sense proteins or their allosteric transitions caused by small molecule bindings, which is to be published separately. Following the same principle, future applications of the technique may also include direct sensing of ribozymes, aptamers, DNA nanostructures^75,76^ or their interactions with small molecules.

## Methods

### Materials

Hexadecane, pentane, ethylenediamine tetraacetic acid (EDTA), Triton X-100, Genapol X-80, calcium chloride (CaCl_2_), tRNA^phe^ from brewer’s yeast, total tRNA from brewer’s yeast and total tRNA from *E.coli* were from Sigma-Aldrich. Dioxane-free isopropyl-β-D-thiogalactopyranoside (IPTG), kanamycin sulfate, imidazole, N,N,N′,N′-tetramethyl-ethylenediamine (TEMED) and tris (hydroxymethyl) aminomethane (Tris) were from Solarbio. DNA Marker DL2000, RNA Marker RL1000, RNA Marker RL6000, RNAiso for Small RNA, MiniBEST Universal RNA Extraction Kit and RNase-free water were from Takara. ZR small-RNA™ PAGE Recovery Kit was from ZYMO research. Low Range ssRNA Ladder was from New England Biolabs. SYBR gold nucleic acid gel stain was from Invitrogen. Potassium chloride (KCl) was from Aladdin. 4-(2-hydroxyethyl)−1-piperazineethanesulfonic acid (HEPES) was from Shanghai Yuanye Biotechnology. 1,2-diphytanoyl-sn-glycero-3-phosphocholine (DPhPC) was from Avanti Polar Lipids. *E. coli* strain BL21 (DE3) was from Biomed. Luria-Bertani (LB) agar and LB broth were from Hopebio. Chloroform was from Labol. Isopropanol and urea were from GHTECH. 75% ethanol (prepared with DEPC treated water) was from KeyGeN. 40% Acrylamide/methylene diacrylamide solution was from Sangon. High-performance liquid chromatography–purified hsa-miR-21, siFoxA1 and luciferase siRNA were hybridized by Sangon and delivered as a double stranded form (Supplementary Table 1).

1.5 M KCl buffer (1.5 M KCl, 10 mM HEPES, pH 7.0), and 1 M CaCl_2_ buffer (1 M CaCl_2_, 10 mM HEPES, pH 7.0) were prepared and membrane-filtered (0.2 μm cellulose acetate; Nalgene) prior to use. RNA was dissolved in RNase-free water before use. The M1 MspA (D90N/D91N/D93N) and M2 MspA (D90N/D91N/D93N/D118R/D134R/E139K) were expressed with *E. coli* BL21 (DE3) and purified by nickel affinity chromatography as described previously^77^. The plasmid DNAs encoding M1 or M2 MspA were custom synthesized by Genescript (New Jersey) and have been shared via https://www.molecularcloud.org/s/shuo-huang. The access codes are MC_0101207 (M1 MspA) and MC_0101191 (M2 MspA). The majority of results were acquired with the M2 MspA. For simplicity, M2 MspA is referred to as MspA throughout the text, if not otherwise stated.

### Nanopore measurements

The measurement device is composed of two custom poly-formaldehyde chambers separated by a ~20 μm-thick Teflon film drilled with an aperture (~100 μm in diameter). Prior to the measurement, the aperture was first treated with 0.5% (v/v) hexadecane (dissolved in pentane) and set for pentane evaporation. Afterwards, 500 μL electrolyte buffers were respectively added to both chambers. A pair of custom Ag/AgCl electrodes, electrically connected to the patch clamp amplifier, were respectively placed in both chambers, in contact with the buffers. Conventionally, the chamber which is electrically grounded was defined as the *cis* chamber, while the opposing chamber was defined as the *trans* chamber. In total 100 µL pentane solution of DPhPC (5 mg/mL) was added to both chambers. A lipid bilayer was formed by pipetting the electrolyte buffer in either chamber up and down several times. Upon the successful formation of the lipid bilayer, the acquired current immediately drops to 0 pA, indicating that the aperture connecting both chambers has been completely sealed. MspA was added to the *cis* chamber to initiate spontaneous pore insertion. Upon a single nanopore insertion, the buffer in the *cis* chamber was manually exchanged to avoid further pore insertions.

To avoid external electromagnetic and vibration noises during the measurements, the device was shielded in a custom Faraday cage (34 cm by 23 cm by 15 cm) mounted on a floating optical table (Jiangxi Liansheng Technology). All electrophysiology measurements were performed with an Axonpatch 200B patch clamp amplifier paired with a Digidata 1550B digitizer (Molecular Devices). All single-channel recordings were sampled at 25 kHz and low-pass filtered with a 1 kHz cutoff frequency. The acquired traces were further digitally filtered with a 500 Hz low-pass Bessel filter (eight-pole) using Clampfit 10.7 (Molecular Devices).

Unless otherwise stated, all nanopore measurements in this paper were performed with a 1.5 M KCl buffer (1.5 M KCl, 10 mM HEPES, pH 7.0) in *cis* and a 1 M CaCl_2_ buffer (1 M CaCl_2_, 10 mM HEPES, pH 7.0) in *trans* and a + 150 mV potential was continuously applied.

### Data analysis

RNA translocation events were recognized with the “single channel research” option in Clampfit 10.7. The machine learning algorithm was custom programmed by Python. Subsequent analyses including histogram plotting and curve fitting were performed in Origin 9.1 (Origin Lab).

### MD simulations

All molecular dynamics (MD) simulations were conducted by GROMACS 2019^78^ with the CHARMM36m force field^79^ and TIP3P water model^80^. The setup of the simulation system was prepared by using the CHARMM-GUI web server^81^. The atomic coordinates of MspA^28^ and tRNA^82^ were taken from the Protein Data Bank (PDB) with the entries 1UUN and 1EVV, respectively. Following the experimental setup, the mutations R96A, D93N, D91N, D90N, D118R, D134R, and E139K were introduced to simulate the composition of an M2 MspA. A 1-palmitoyl-2-oleoyl-glycero-3-phosphocholine (POPC) lipid bilayer with the size of 12 × 12 nm^2^ was added. The resulting system was then solvated in a rectangular water box with periodic boundary condition. To simplify the simulations, the system was established in a symmetric KCl buffer electrolyte system. K^+^ and Cl^−^ ions were added at random positions to give a salt concentration of 1.5 M and to neutralize the simulation system. The final system consists of ~225,000 atoms. The long-range electrostatic interactions were calculated using the smooth particle-mesh Ewald method^83^. The cutoff distance for the calculations of the short-range part of the electrostatic interactions and the van der Waals interactions were set to 1.2 nm. The covalent bonds involving hydrogen atoms were restrained with the LINCS algorithm^84^.

To simulate tRNA translocation, each system was firstly minimized for 1000 steps and then equilibrated at 298 K for 0.25 ns under NVT ensemble using Berendsen weak-coupling thermostat^85^. The heated systems were further equilibrated under NPT ensemble at 298 K and 1 atm for another 1.75 ns, with the Berendsen semi-isotropic barostat^85^, leading to a box size of ~11.6 nm × 11.6 nm × 16.5 nm. The simulations of translocation were initiated from the final structures of the above equilibrating simulations with NVT ensemble. An external electric field of 2.0 V/10 nm was applied along the direction perpendicular to the membrane plane for 0.5 ns, then the external electric field was switched to 4.0 V/10 nm. The production simulations lasted for 100 ns with a time step of 2 fs. During the simulations, harmonic positional restraints were applied to the C_α_ atoms of MspA with a spring constant of 500 kJ/mol/nm^2^. Experimentally, translocation of tRNA typically lasts ~second, which is far beyond the accessible timescale of conventional all-atom MD simulations. In order to observe a full translocation process within a feasible simulation timescale, the external electric field of 4.0 V/10 nm used in the translocation simulations corresponds to a much higher voltage bias than that is applied in the experiment. As discussed in previously reported literatures^86,87^, high electric fields often result in the formation of electroporation of the lipid bilayer even in short MD simulations, which can lead to ion leakages. Consequently, different simulation strategies were used to avoid the formation of electroporation of the lipid bilayer, such as adding positional restraints^88^, using puling force with steered MD to drive the translocation^87,89^, or using more sophisticated Grid-steered MD^90^. Here we applied positional restraints to avoid the formation of electroporation, in which all the heavy atoms of the lipid molecules were restrained to the positions in the structures obtained from the minimization step by a harmonic potential with a spring constant of 1000 kJ/mol/nm^2^.

To characterize the simulated tRNA translocation process, we used three reaction coordinates, including the number of base-pair hydrogen bonds (H-bond), the root mean square deviation (RMSD) from the native structure, and the z-coordinate of the tRNA (Z). The H-bond represents the number of hydrogen bonds between the nucleotide pairs which form base pairs in the native structure. Therefore, decrease of the H-bond corresponds to the disruption of the tRNA base pairing. The RMSD characterizes the overall structure change of the tRNA, which is not only sensitive to the structural unfolding, but is also sensitive to the overall deformation of the molecules. Therefore, the H-bond and RMSD can be applied to describe different conformational properties of the tRNA during translocation. The reaction coordinate Z is defined by the z-coordinate of the leading nucleotide during the translocation (green sphere in Fig. 4a–c). The nucleotides A76, G34, or U55 were respectively assigned as the leading nucleotides for simulations with the stem-down, the loop-down, and the arm-down orientations. Z = 0 corresponds to the z position of the narrowest spot in the MspA pore (Fig. 4a–c), which was defined by the center of mass of the ${{\rm{C}}}_{{\rm{\alpha }}}$ atoms of the N90 of all eight subunits. Z < 0 means that the leading nucleotide has successfully translocated through the pore.

To simulate the ionic current, starting from the equilibrated structures with the above-mentioned three different tRNA orientations, the systems were firstly relaxed for 20 ns under an external electric field of 1.0 V/10 nm, so that the tRNA makes sufficient contacts with the entrance of the MspA. The production simulations started from the relaxed structures under an external electric field of 0.09 V/10 nm, which corresponds to a voltage bias of ~+150 mV, similar to that used in the experiments. The production simulations lasted for 100 ns. We also repeated the simulations at higher electric fields, including 0.2 V/10 nm and 0.6 V/10 nm. As the lipid bilayer can keep stable under these electric fields within the simulation timescale, the positional restraints were applied only to the C_α_ atoms of the MspA and the lipid molecules are free to move. Following a previous study^57^, the instantaneous ionic current was calculated based on the coordinates of the ions. Since the instantaneous ionic current has large fluctuations, we calculated the cumulative currents. The ionic currents were derived from the slope of the cumulative currents by linear fitting. In addition to the above-mentioned three simulation systems, we also performed ionic current simulations for the systems without the presence of any tRNA and the state when the tRNA is translocating through the pore (*Z* < 0). The initial structure of the system with the tRNA translocating through the pore was extracted from the above translocation simulations. The software PyMOL was used for the structural visualization^91^.

Similar simulations were performed for translocation of 5 S rRNA (Supplementary Fig. 25). The POPC lipid bilayer has the size of 13 × 13 nm^2^. The atomic coordinates of 5 S rRNA were taken from the PDB with the entry 1C2X. The final system for the translocation of the 5 S rRNA consists of ~270,000 atoms with a box size of ~12.5 nm × 12.5 nm × 17.0 nm.

### RNA recovery from polyacrylamide gels

In total 30 μg commercial *E.coli* total tRNA (Sigma-Aldrich) was loaded into a 12% denaturing urea polyacrylamide gel. Gel electrophoresis was continuously run for 100 min with a + 180 V applied potential. The gel was illuminated with a portable UV lamp (254 nm). The gel fragments respectively containing 5 S rRNA or tRNA were excised for further recovery. RNA recovery was performed using ZR small-RNA™ PAGE Recovery Kit (ZYMO research). According to the manual, crushed RNA fragment was transferred into a Zymo-Spin™ IV Column. In total 400 μL RNA Recovery Buffer was added to the column and incubated at 65 °C for 15 min. The column was quickly frozen in a −80 °C freezer for 5 minutes and incubated at 65 °C for 5 min. The column was then centrifuged at 1500 × *g* for 30 s. The filtrate was transferred to a Zymo-Spin™ IIICG Column and centrifuged at 1500 × *g* for 30 s. The filtrate was added with 2 volumes of RNA MAX Buffer and thoroughly mixed. The mixture was then transferred to a Zymo-Spin™ IC Column and centrifuged at 12000 × *g* for 30 s and the supernatant was discarded. The column was added with 400 µL RNA Prep Buffer and centrifuged at 12,000 × *g* for 1 min and the filtrate was discarded. The column was added with 800 µL RNA Wash Buffer and centrifuged at 12,000 × *g* for 1 min then the filtrate was discarded. The column was added with 400 µL RNA Wash Buffer and centrifuged at 12,000 × *g* for 1 min and the filtrate was discarded. The column was centrifuged at 12,000 × *g* for 2 min to ensure complete removal of the wash buffer. The column was added with 30 µL RNase-free water. After standing for 1 min, the column was centrifuged at 10,000 × *g* for 1 min to elute the RNA. The eluted RNA concentration was determined by nanodrop (Thermo, USA) and the sample was further characterized using 12% denaturing urea polyacrylamide gel electrophoresis. Finally, the recovered RNA was stored at −80 °C for subsequent electrophysiology measurements. All tips and tubes used were RNase-free.

### LMW RNA extraction from *E.coli*

*E. coli* strain BL21 (DE3) was cultured in LB broth and shaken overnight (230 × rpm) at 16 °C. The cells were pelleted by centrifugation at 12,000 × *g* for 20 min at 4 °C and washed with 1× PBS to remove residual LB broth. The deposition was collected and lysed in 1 mL RNAiso for Small RNA (Takara). After vigorous vortexing, the lysis solution was placed at room temperature (rt) for 5 min. To extract LMW RNA, the lysis solution was added with 200 μL chloroform and fully emulsified through vortexing. After standing for 5 min, the mixture was centrifuged at 12,000 × *g* for 15 min at 4 °C. When carefully removed from the centrifuge, the mixture was divided into three layers: the colorless supernatant containing LMW RNA, the white middle layer containing protein and the colored lower layer containing the organic solvent. The supernatant was transferred to a new centrifugal tube and added with 600 μL isopropanol. After thorough mixing, it was set for 10 min at 15–30 °C. The mixture was centrifuged at 12,000 × *g* for 10 min at 4 °C to collect the pellet. The pellet was washed with 1 mL 75% ethanol and centrifuged at 12,000 × *g* for 5 min at 4 °C and the supernatant was discarded. The pellet, which is the LMW RNA, was dried at room temperature for 30 min. A total of 25 μL of RNase-free water was then added to dissolve the LMW RNA. The concentration of the sample was determined by nanodrop. This LMW RNA sample was further characterized using 12% denaturing urea polyacrylamide gel electrophoresis. Finally, LMW RNA was stored at −80 °C for subsequent electrophysiology measurements. All tips and tubes used are RNase-free.

### HMW RNA extraction from *E.coli*

High molecular weight (HMW) RNA ( > 200 nt) of *E.coli* (BL21) DE3 was extracted using MiniBEST Universal RNA Extraction Kit. *E. coli* strain BL21 (DE3) was cultured in LB broth and shaken overnight (230 rpm) at 16 °C. The cells were pelleted by centrifugation at 13,800 × *g* for 20 min at 4 °C and washed with 1× PBS water to remove residual LB broth. A total of 350 μL lysis Buffer RL was added to the collected cells. The lysate was transferred to a gDNA Eraser Spin Column and centrifuged at 13,800 × *g* for 1 min at 20 °C to remove the gDNA. The filtrate was added with isopycnic 70% ethanol and mixed thoroughly. The mixture was transferred to RNA Spin Column and centrifuged at 13800 × *g* for 1 min at 20 °C. The RNA Spin Column was added with 500 μL Buffer RWA and centrifuged at 13,800 × *g* for 30 s at 20 °C. The filtrate was discarded. The RNA Spin Column was added with 600 μL buffer RWB and centrifuged at 13800 × *g* for 3 min at 20 °C. The RNA Spin Column was placed onto 1.5 mL RNase Free Collection Tube and added with 30–200 μL RNase free water. After 5 min, HMW RNA was eluted by centrifugation at 13,800 × *g* for 2 min at 20 °C. The concentration was measurement using nanodrop and the desired fraction was determined using 1% agarose gel electrophoresis. Finally, HMW RNA was stored at −80 °C for subsequent electrophysiology measurements. Tips and tubes used were RNase-free.

### Reporting summary

Further information on research design is available in the Nature Research Reporting Summary linked to this article.

## Supplementary information

Supplementary Information

Reporting Summary

Description of Additional Supplementary Files

Supplementary Movie 1

Supplementary Movie 2

Supplementary Movie 3

Supplementary Movie 4

Supplementary Movie 5

## Data Availability

The datasets generated during and/or analyzed during the current study are available from the corresponding author on reasonable request.

## Code availability

The machine learning based executable software “*RNA-Classification*” and its code have been deposited at https://drive.google.com/file/d/17JoqS2JUY-Q0Y4e5Ib0HE4PsexYtEIKq/view?usp=sharing. The workflow of this software is provided in Supplementary Fig. 31. A set of demo events were accompanied for code validation. All data presented in this work can be provided by the corresponding authors upon reasonable requests.
