# Supplementary material for: Structural-profiling of low molecular weight RNAs by nanopore trapping/translocation using Mycobacterium smegmatis porin A
Source: Nat Commun. 2021 Jun 7;12:3368. doi: 10.1038/s41467-021-23764-y (PMC8185011; doi:10.1038/s41467-021-23764-y)
Supplement: Supplementary file 1 — Supplementary Information [file 41467_2021_23764_MOESM1_ESM.pdf]

Supporting Information

Structural-profiling of low molecular weight RNAs by nanopore trapping/translocation using *Mycobacterium smegmatis* porin A

Yuqin Wang[1,2], Xiaoyu Guan[3], Shanyu Zhang[1,2], Yao Liu[1,2], Sha Wang[1,2], Pingping Fan[1,2], Xiaoyu Du[1,2], Shuanghong Yan[1,2], Panke Zhang[1], Hong-Yuan Chen[1], Wenfei Li[4*], Daoqiang Zhang[3*] and Shuo Huang[1,2*]

[1.] State Key Laboratory of Analytical Chemistry for Life Sciences, School of Chemistry and Chemical Engineering, Nanjing University, 210023, Nanjing, China.

[2.] Chemistry and Biomedicine Innovation Center (ChemBIC), Nanjing University, 210023, Nanjing, China.

[3.] College of Computer Science and Technology, Nanjing University of Aeronautics and Astronautics, MIIT Key Laboratory of Pattern Analysis and Machine Intelligence

[4.] Collaborative Innovation Center of Advanced Microstructures, National Laboratory of Solid State Microstructure, Department of Physics, Nanjing University, Nanjing 210093, China.

These authors contributed equally: Yuqin Wang, Xiaoyu Guan

These authors jointly supervised this work: Wenfei Li, Daoqiang Zhang and Shuo Huang

Email: wfli@nju.edu.cn (W.F.L.), dqzhang@nuaa.edu.cn (D.Q.Z.), shuo.huang@nju.edu.cn (S.H.)

Supplementary Table 1: Nucleic acid abbreviations and sequences.

abbreviations	sequences (5'-3')
hsa-miR-21	UAGCUUAUCAGACUGAUGUUGA
siFoxA1-a	CUUACGCUGAGUACUUCGAAA
siFoxA1-b	UCGAAGUACUCAGCGUAAGUG
luciferase siRNA-a	AGCAAUAGUUCACGCUGAAAG
luciferase siRNA-b	CUUUCAGCGUGAACUAUUGCU

Footnote: SiFoxA1 was prepared by the hybridization of siFoxA1-a and siFoxA1-b. Luciferase siRNA was prepared by the hybridization of luciferase siRNA-a and luciferase siRNA-b.

Supplementary Table 2: Statistics for hsa-miR-21 sensing in different buffers. All measurements were performed as described in Methods. Hsa-miR-21 was added to *cis* with a final concentration of 200 nM. I_p was derived from Gaussian fitting results. τ_{off} and τ_{on} were derived from single exponential fitting results. All statistical results were from 5-min continuous recordings for each condition. $\overline{I_p}$, $\overline{\tau_{off}}$ and $\overline{\tau_{on}}$ were mean values of I_p, τ_{off} and τ_{on} from three independent measurements, respectively.

cis	*trans*	$\overline{I_p}$	$\overline{\tau_{off}}$/ms	$\overline{\tau_{on}}$/ms
1.5 M KCl	1.5 M KCl	0.847 ± 0.006	1.02 ± 0.14	470 ± 120
1.5 M KCl	1.0 M $CaCl_2$	0.976 ± 0.001	4.2 ± 0.4	165 ± 12
1.0 M $CaCl_2$	1.0 M $CaCl_2$	0.980 ± 0.000	4.5 ± 1.1	350 ± 80
1.0 M $CaCl_2$	1.5 M KCl	0.933 ± 0.005	2.5 ± 0.3	1300 ± 300

Supplementary Table 3: I_p of RNA sensing by MspA. All measurements were performed as described in Methods. I_p was derived from Gaussian fitting results. $\overline{I_p}$ was the mean value of I_p from three independent measurements.

types of RNA	$\overline{I_p}$
miRNA	0.976 ± 0.001
overhanged siRNA	0.600 ± 0.006
blunt siRNA	0.490 ± 0.010 (type 1)/0.533 ± 0.004 (type 2)
tRNA	0.567 ± 0.004 (type 1)/0.453 ± 0.002 (type 2)
5S rRNA	0.356 ± 0.003 (type 1)/0.566 ± 0.017 (type 2)/0.737 ± 0.005(type 3)

Supplementary Table 4: τ_{off} of tRNA^{phe} measured at different voltages. All measurements were performed as described in Methods. tRNA^{phe} was added to the *cis* chamber with a final concentration of 200 nM. τ_{off} was derived from single exponential fitting results. $\overline{\tau_{off}}$ was the mean value of τ_{off} from three independent measurements.

voltage/mV	$\overline{\tau_{off}}$ of type 1 /ms	$\overline{\tau_{off}}$ of type 2 /ms
125	900 ± 800	82500 ± 800
150	3000 ± 2000	1600 ± 500
175	11900 ± 500	1290 ± 90
200	20000 ± 2000	980 ± 130
225	22000 ± 2000	700 ± 200

Supplementary Table 5: I_p of tRNA events from different biological sources. All measurements were performed as described in Methods. tRNA^{phe} was added to *cis* with a final concentration of 200 nM. Yeast total tRNA was added to *cis* with a final concentration of 20 ng/µL. *E.coli* total tRNA (unpurified) was added to *cis* with a final concentration of 20 ng/µL. *E.coli* total tRNA (purified) was added to *cis* with a final concentration of 2 ng/µL. I_p was derived from Gaussian fitting results. $\overline{I_p}$ was the mean value of I_p from three independent measurements.

biological samples	$\overline{I_p}$_type 1 level 1	$\overline{I_p}$_type 2 level 1	$\overline{I_p}$_type 2 level 2
tRNA^{phe}	0.567 ± 0.004	0.453 ± 0.002	0.970 ± 0.003
Yeast total tRNA	0.549 ± 0.009	0.451 ± 0.002	0.979 ± 0.012
E.coli total tRNA (purified)	0.55 ± 0.02	0.478 ± 0.004	0.986 ± 0.002

Supplementary Table 6: Proportion of tRNA signals determined with machine learning algorithms. All measurements were performed as described in Methods. Different analytes were respectively added to *cis*. Three independent measurements were performed for each condition to produce the statistics.

biological samples	proportion of tRNA signals	proportion of type 1 signals	proportion of type 2 signals
tRNA^{phe}	0.71 ± 0.05	0.41 ± 0.05	0.30 ± 0.04
Yeast tRNA	0.380 ± 0.013	0.21 ± 0.04	0.17 ± 0.03
E.coli tRNA (purified)	0.402 ± 0.009	0.206 ± 0.016	0.196 ± 0.007
E.coli LMW RNA	0.48 ± 0.11	0.24 ± 0.09	0.23 ± 0.06

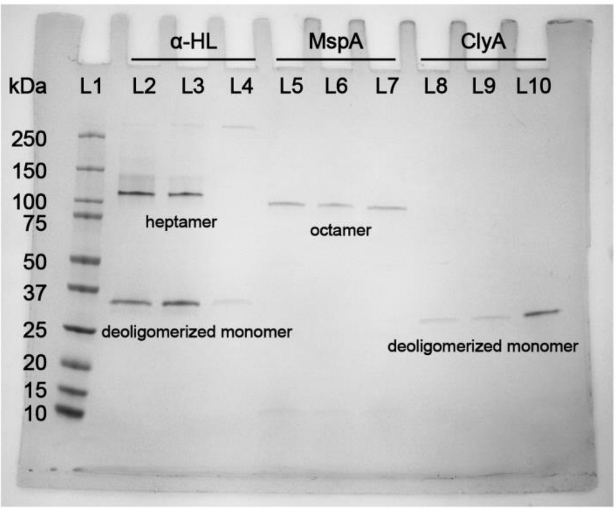

Supplementary Figure 1: Stability comparison between different protein nanopores. L1: protein marker (precision plus protein standards, BIO-RAD, USA); L2: freshly prepared WT α-HL heptamer; L3: WT α-HL heptamer treated at 80 °C for 15 minutes; L4: WT α-HL heptamer stored at -80 °C for 3 months; L5: freshly prepared M2 MspA octamer; L6: M2 MspA octamer treated at 80 °C for 15 minutes; L7: M2 MspA octamer stored at -80 °C for 3 months; L8: freshly prepared ClyA-RR; L9: ClyA-RR treated at 80 °C for 15 minutes; L10: ClyA-RR stored at -80 °C for 3 months. The M2 MspA demonstrated the best stability among all three nanopores by showing an unaltered octameric assembly form; A fraction of the freshly prepared WT a-HL heptamer showed deoligomerization when heated or stored for 3 months; ClyA-RR showed complete deoligomerization under the condition of SDS-PAGE.

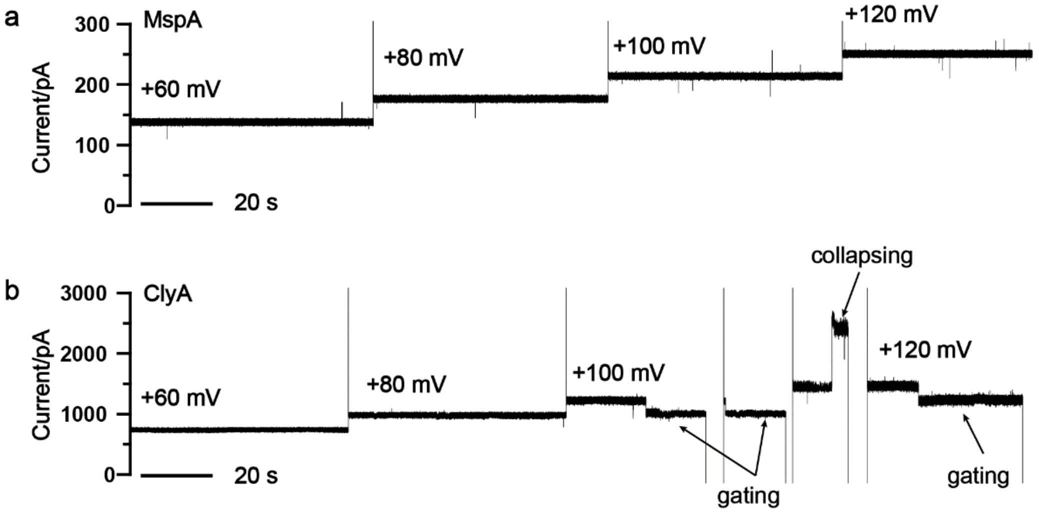

Supplementary Figure 2: Comparison of high voltage performance between M2 MspA and ClyA-RR. Continuous long-term measurements with MspA at a high applied potential show stable open pore current (**a**). Based on previously reported literatures, the M2 MspA can sustain long term measurements at an applied potential up to +200 mV. On the other hand, the performance of ClyA-RR is much worse (**b**). Appearance of spontaneous gating or pore collapsing were frequently observed when the applied potential is more than +100 mV. Electrophysiology measurements were performed as described in Methods. An electrolyte buffer of 1.5 M KCl (*cis*)/ 1 M $CaCl_2$ (*trans*) as applied.

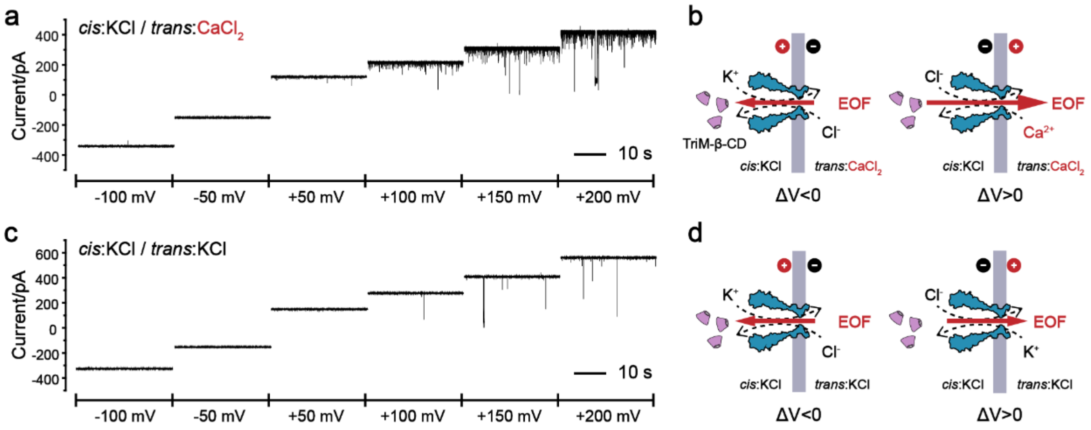

Supplementary Figure 3: Experimental evidence of EOF in MspA. Similarly to previous studies[1, 2], EOF in MspA (blue) was determined by observing the voltage-dependence of events generated by neutral analytes. Trimethyl-β-cyclodextrin (TriM-β-CD, purple) was applied as the analyte. **a.** Voltage dependence of TriM-β-CD sensing using MspA when a 1.5 M KCl buffer and a 1 M $CaCl_2$ buffer were separately added to *cis* and *trans*. The rate of event appearance increased at a higher applied potential. No events were observed at negative potentials. It implies that an EOF from *cis* to *trans* exists in MspA when a potential was applied. **b.** Cartoon diagrams of the EOF flow in MspA at negative (left) and positive (right) potentials. The direction of EOF was indicated with the red arrows. **c.** Voltage dependence of TriM-β-CD sensing using MspA in a symmetric 1.5 M KCl buffer. The rate of event appearance was lower than that demonstrated in (**a**). However, slightly more events were observed at a higher potential, indicating the existence of a weak EOF. **d**. Cartoon diagrams of the EOF flow in MspA at negative (left) and positive (right) potentials. The direction of EOF was indicated with the red arrows. The measurements in (**a, c**) were performed as described in Methods**.** The applied potential was indicated in the x axis of (**a, c**). TriM-β-CD was added to *cis* with a final concentration of 2 mM.

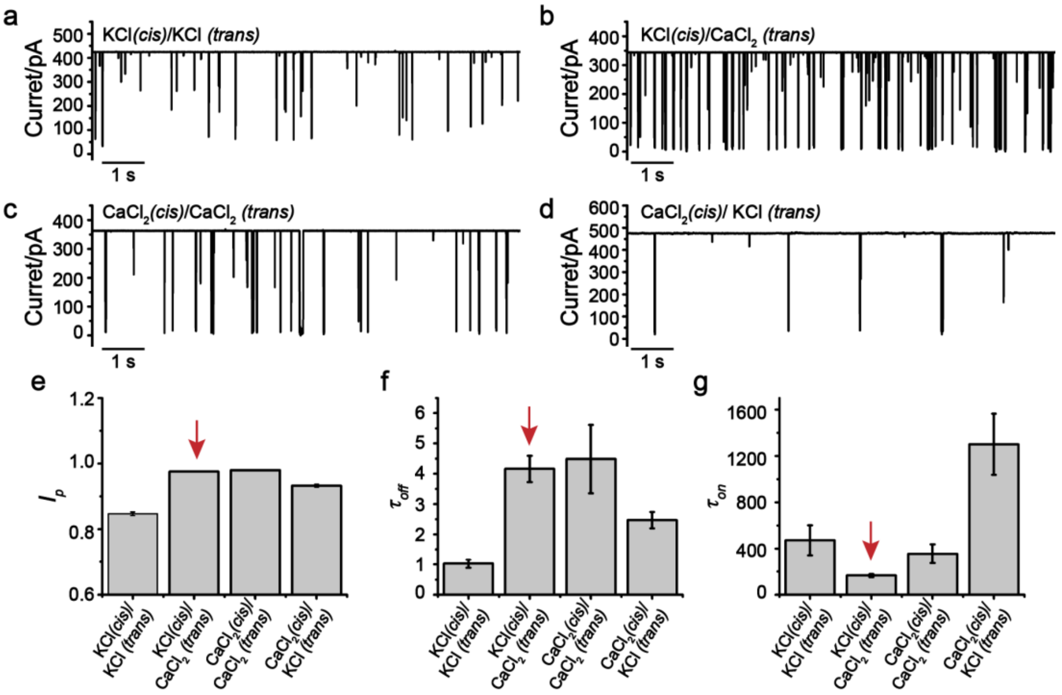

Supplementary Figure 4: miRNA sensing with varied salt combinations. a-d. Representative traces of miRNA sensing performed with a buffer of 1.5 M KCl (*cis*)/1.5 M KCl (*trans*) (**a**), 1.5 M KCl (*cis*)/1 M $CaCl_2$ (*trans*) (**b**), 1 M $CaCl_2$ (*cis*)/1 M $CaCl_2$ (*trans*) (**c**) or 1 M $CaCl_2$ (*cis*)/1.5 M KCl (*trans*) (**d**). **e.** I_p of miRNA events with varied salt combinations. **f.** τ_{off} of miRNA events with varied salt combinations. **g.** τ_{on} of miRNA events with varied combinations. The red arrows indicate the asymmetric KCl/$CaCl_2$ buffer condition we used in our manuscript. Error bars in (E-F) represent standard deviation, n = 3 independent replicates. Has-miR-21 was added to *cis* with a final concentration of 200 nM. A voltage of +150 mV was continuously applied during the measurements.

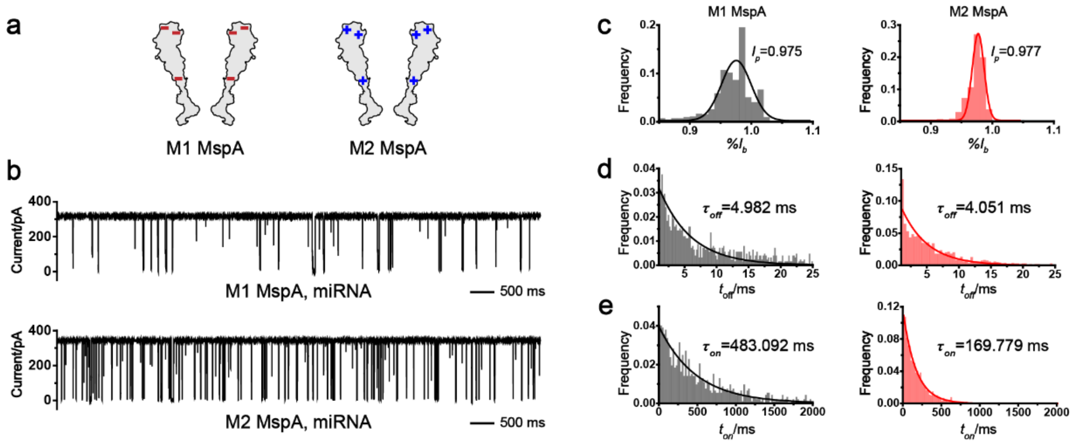

Supplementary Figure 5: miRNA sensing with M1 or M2 MspA. a. The internal charge distribution of MspA mutants (the M1 or M2 MspA). **b**. Representative current traces of miRNA translocations through an M1 or M2 MspA. **c**. Histogram of miRNA translocation events performed with M1 (grey) or M2 MspA (red). **d.** Histogram of t_{off} of miRNA translocation events performed with M1 (grey) or M2 MspA (red). **e**. Histogram of t_{on} of miRNA translocation events performed with M1 (grey) or M2 MspA (red). Due to the presence of positively charged amino acids in the pore lumen, the M2 MspA shows a higher capture rate of RNA molecules. Electrophysiology measurements were performed in 1.5 M KCl (*cis*)/ 1 M $CaCl_2$ (*trans*). MiRNA was added to *cis* with a final concentration of 200 nM. A voltage of +150 mV was continuously applied during the measurements.

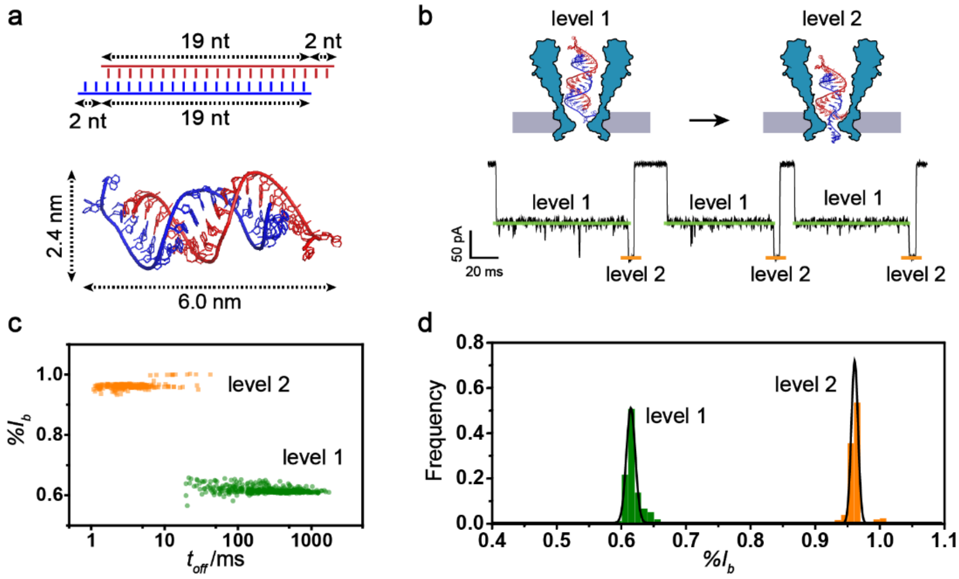

Supplementary Figure 6: Overhanged siRNA translocation events. a. The structure of overhanged siRNA (PDB: 1RUP). The secondary structure of overhanged siRNA (top) is composed of a 19-bp double strand and two 2-nt overhangs. They fold into an A-form duplex (bottom). **b.** Representative overhanged siRNA translocation events (bottom) and a suggested model of translocation (top). The model suggests that overhanged siRNA first partially blocks the pore followed with an electrophoretically driven unzipping of the duplex. The measurement was performed with a +150 mV continuously applied potential. In this condition, the majority of overhanged siRNA translocation events appear as characteristic 2-step shaped events (bottom left), which start with an initial partial blockage (level 1) followed with a further deeper blockage (level 2). Please note that some events may have missing level 1 or level 2. Events with missing level 1 is however extremely rare to observe. Events with missing level 2 are well recognized since the characteristics of level 1 is more significant in the evaluation of the event identity. **c.** The scatter plot of $\%I_b$ versus t_{off}. **d.** The corresponding event histogram of $\%I_b$. Nanopore measurements were performed as described in Methods. Hybridized overhanged siFoxA1 (Materials, Supplementary Table 1) was added to *cis* with a final concentration of 200 nM.

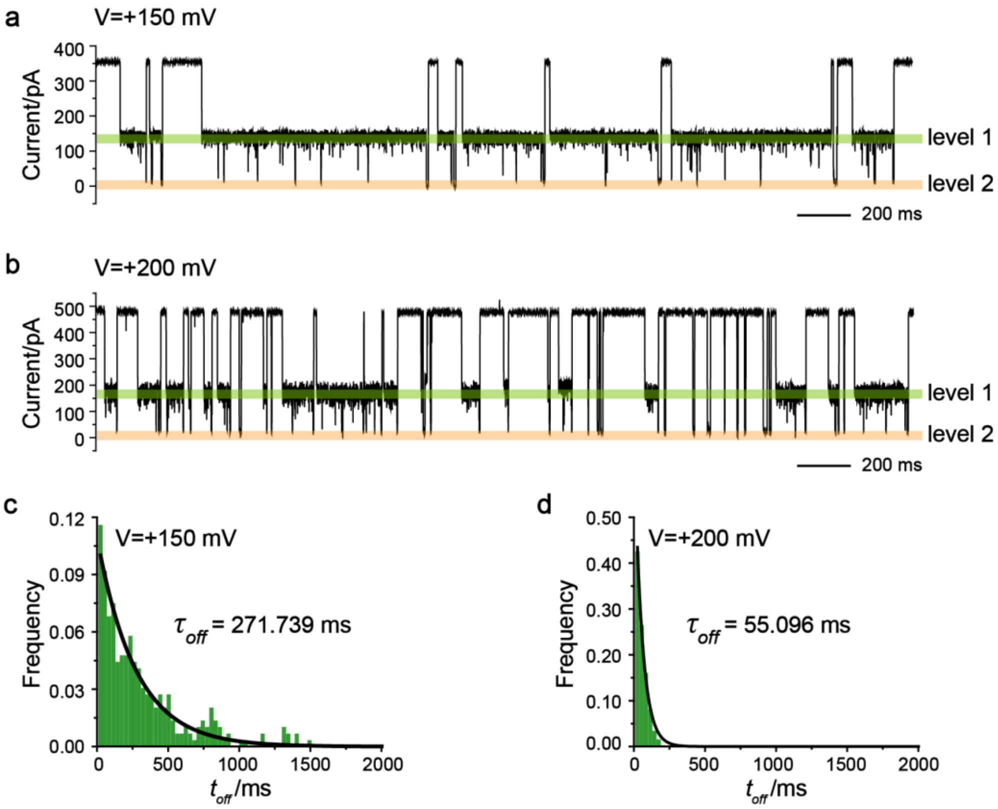

Supplementary Figure 7: Overhanged siRNA translocation events at different voltages. a. A representative trace of overhanged siRNA translocation acquired at +150 mV. **b.** A representative trace of overhanged siRNA translocation acquired at +200 mV. With a strong electrophoretic force applied, events caused by overhanged siRNA appears with a significantly reduced dwell time of level 1, indicating that the analyte has translocated through the pore. **c.** The event histogram of t_{off} of level 1 at +150 mV. **d.** The event histogram of t_{off} of level 1 at +200 mV. Nanopore measurements were performed as described in Methods. Hybridized overhanged siFoxA1 (Materials, Supplementary Table 1) was added to *cis* with a final concentration of 200 nM.

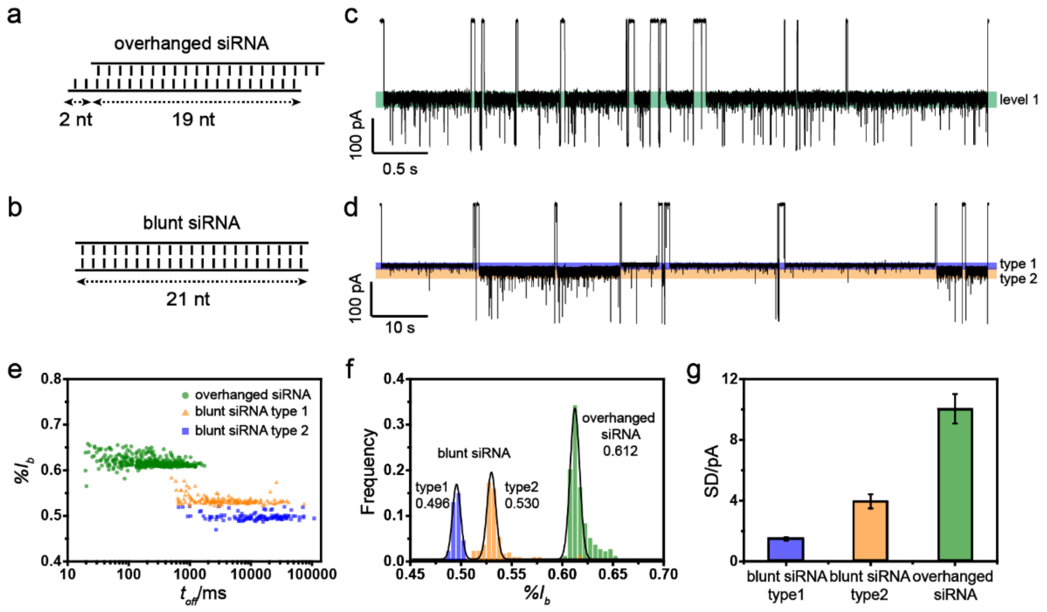

Supplementary Figure 8: Discrimination of overhanged and blunt siRNA with MspA. a-b. The secondary structures of overhanged and blunt siRNA. **c**. Representative trace of overhanged siRNA sensing with MspA. Characteristic events appear as a two-step blockade. **d**. Representative trace of blunt siRNA sensing with MspA. Two types of signals with different blockade amplitudes are observed. **e**. A scatter plot of t_{off} versus $\%I_b$ for two RNA samples. Events from two types of RNAs are clearly distinguishable. **f**. The corresponding event histogram of $\%I_b$ of two RNA samples. Black lines are Gaussian fittings to the data. **g**. Standard deviation of characteristic levels (marked in **d** and **e**) for two RNA samples. Error bars represent standard deviation. 20 events of each types were analyzed to form the statistics. Electrophysiology measurements were performed in 1.5 M KCl (*cis*)/ 1 M $CaCl_2$ (*trans*). RNA was added to *cis* with a final concentration of 200 nM. A voltage of +150 mV was applied during the measurements.

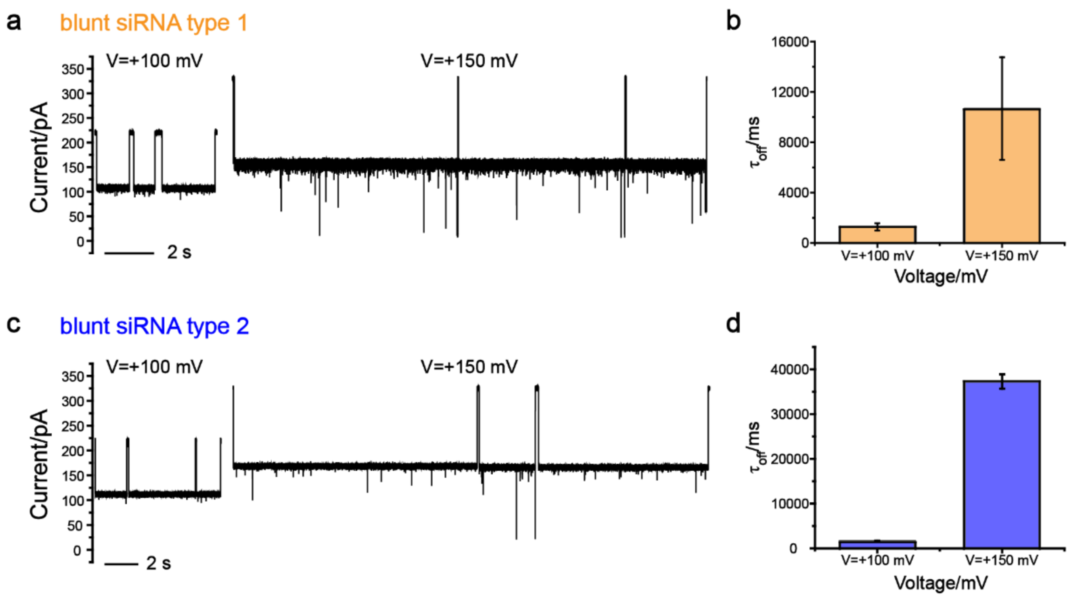

Supplementary Figure 9: Blunt siRNA translocation events at different voltages. a. Representative type 1 events at +100 mV and +150 mV. The event dwell time is extended when the applied potential is increased. **b.** τ_{off} of type 1 events at different voltages. **c.** Representative type 2 events at +100 mV and +150 mV. The dwell time increases when the applied potential is increased. **d.** τ_{off} of type 2 events at different voltages. The reported events result from nanopore trapping instead of translocation. All measurements were carried out as described in Methods. Blunt siRNA was added to *cis* with a final concentration of 200 nM. Error bars in (**b**) and (**e**) represent standard deviation, n = 3 independent replicates.

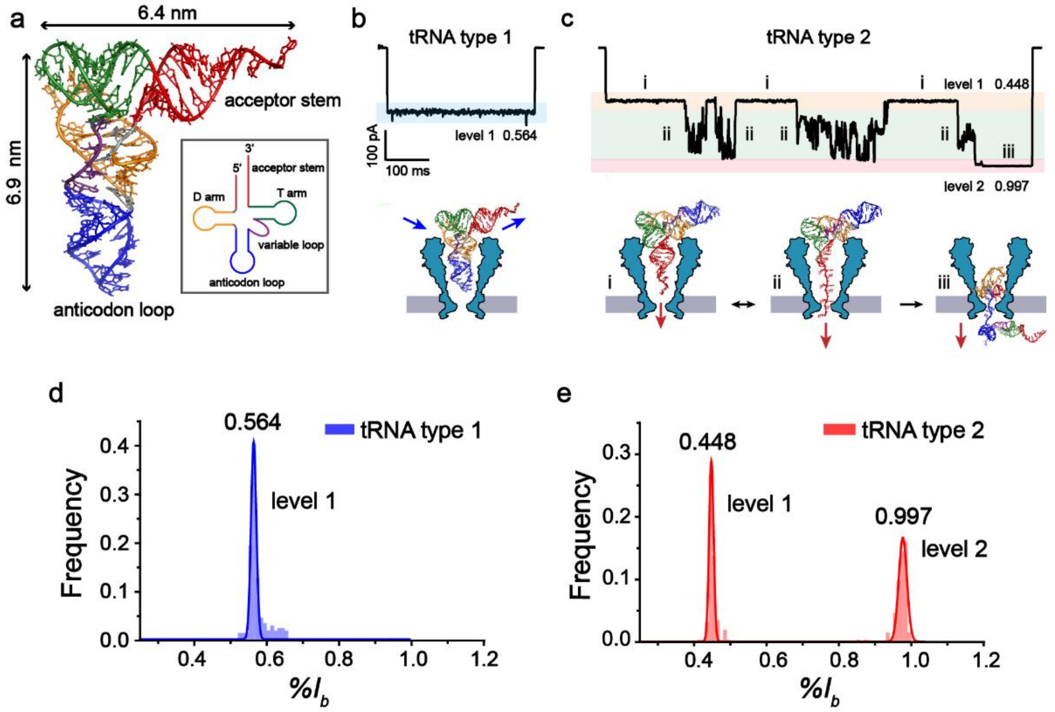

Supplementary Figure 10: Translocation of yeast tRNA^{phe}. a. The structure of yeast tRNA^{phe}. The secondary structure of yeast tRNA^{phe} (shown in the rectangular box) is composed of four domains, including the acceptor stem (red), the D-arm (orange), the T-arm (green) and the anticodon loop (blue). They fold into an L-shaped tertiary structure, where the anticodon loop and the acceptor stem are located at the two ends of the "L" shape. **b.** A representative tRNA type 1 event (top) and the diagram of a possible configuration of translocation (bottom). The type 1 event has a single blockage level with a $\%I_b$ of about 0.564 (level 1). **c.** A representative tRNA type 2 event (top) and the diagram of a possible translocation configuration (bottom). The type 2 event has two blockage levels. The $\%I_b$ measures about 0.448 (i, level 1). Triggered by the applied electrophoretic force, the overhang of the acceptor stem attempts to enter the pore constriction, producing drastic current fluctuations (ii). tRNA^{phe} is eventually unfolded which gives rise to the second blockage level with a $\%I_b$ of about 0.997 (iii, level 2). **d.** The histogram of $\%I_b$ of type 1 events. **e.** The histogram of $\%I_b$ of type 2 events. Nanopore measurements were performed as described in Methods**.** tRNA^{phe} (Materials, Supplementary Table 1) was added to *cis* with a final concentration of 200 nM.

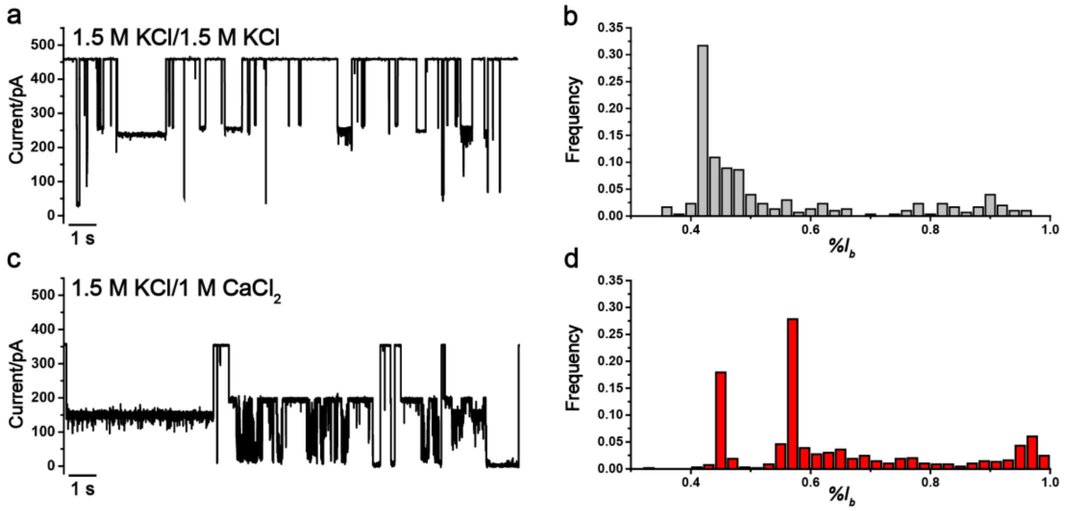

Supplementary Figure 11: tRNA^{phe} sensing with different buffers. Nanopore measurements were performed as described in Methods. The electrolyte buffer in *trans* is either a 1.5 M KCl buffer (1.5 M KCl, 10 mM HEPES, pH 7.0) or a 1 M $CaCl_2$ buffer (1 M $CaCl_2$, 10 mM HEPES, pH 7.0). tRNA^{phe} was added to *cis* with a 200 nM final concentration. **a.** A representative trace containing successive tRNA^{phe} translocation events. A 1.5 M KCl buffer was employed in both *cis* and *trans*. The majority of translocation events has only one step and does not appear uniformly. **b.** The event histogram of $\%I_b$ from a time extended measurement as described in (**a**). In this condition, $\%I_b$ is widely distributed. **c.** A representative trace containing successive tRNA^{phe} translocation events. A 1.5 M KCl buffer in *cis* and a 1 M $CaCl_2$ buffer in *trans* was employed. The dwell time of tRNA events were significantly extended and the characteristic tRNA translocation events (Fig. 2b) were frequently observed. **d.** The event histogram of $\%I_b$ of event level 1 (Fig. 2b) from a time extended measurement as described in (**c**). From the histogram, characteristic two populations of events were clearly observable. Based on previously reported literatures[3-5] and our results, we speculate that the presence of Ca^{2+} is two-fold, to retard the translocation of the analyte and to maintain the structure of the tRNAs. Though not tested in this work, other divalent ions such as Mg^{2+} may have similar effects stabilizing tRNA structures.

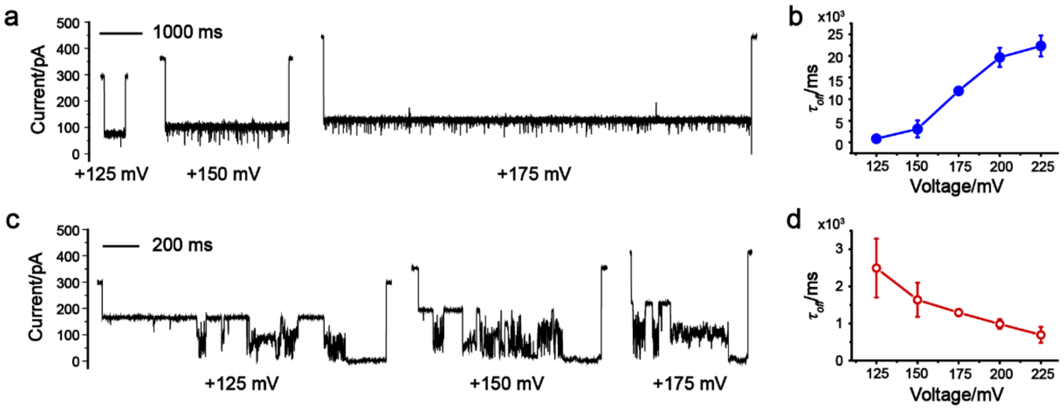

Supplementary Figure 12: tRNA^{phe} translocation events at different voltages. a. Representative type 1 events at +125 mV, +150 mV and +175 mV. The event dwell time is extended when the applied potential is increased. **b.** τ_{off} of type 1 events at different voltages. **c.** Representative type 2 events at +125 mV, +150 mV and +175 mV. The dwell time decreases when the applied potential is increased. **d.** τ_{off} of type 2 events at different voltages. All measurements were carried out as described in Methods. tRNA^{phe} was added to *cis* with a final concentration of 200 nM. Error bars in (**b**) and (**d**) represent standard deviation, n = 3 independent replicates.

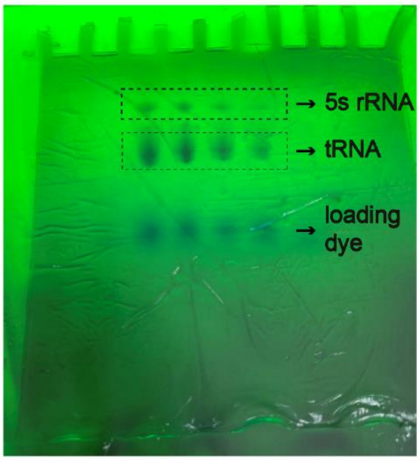

Supplementary Figure 13: Preparation of *E.coli* 5S rRNA. *E.coli* low molecular weight (LMW) RNA (<200 nt) extracted by the small RNA extraction reagent from Takara (Methods), was loaded onto a 12% urea-PAGE gel. Gel electrophoresis was continuously run for 100 min with a +180 V applied potential. The gel was visualized with a portable UV lamp (254 nm). Three bands were clearly observable and were respectively recognized as 5S rRNA, tRNA and xylene cyanol, according to the published literature[6]. The region corresponding to 5S rRNA was separately excised. The excised gel fragments were treated with the ZR small-RNA™ PAGE Recovery Kit (ZYMO Research, USA) to recover the RNA (Methods).

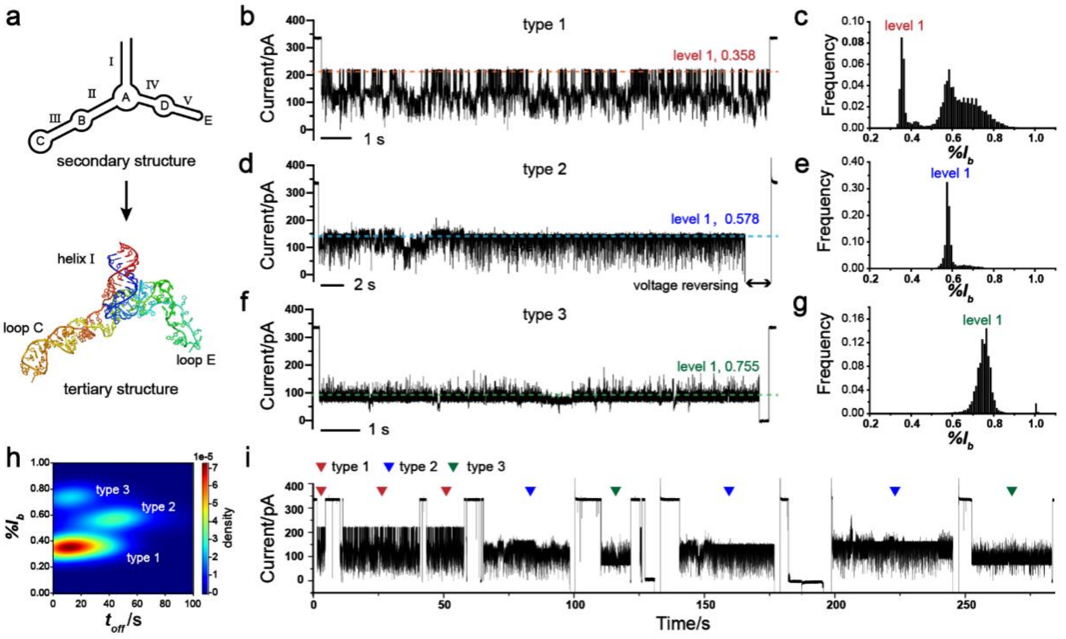

Supplementary Figure 14: **Translocation of *E.coli* 5S rRNA. a.** The structure of *E.coli* 5S rRNA (PDB: 1C2X). The secondary structure of *E.coli* 5S rRNA (top) is composed of five helices (denoted I-V in roman numerals), four loops (B-E), and one hinge (A), which form together a Y-like tertiary structure (bottom). The loop C, loop E and helix I are located at the three ends of the "Y" shape. **b.** A representative 5S rRNA type 1 event. The type 1 event appears as reciprocating current oscillations below a constant blockade with a $\%I_b$ of about 0.358 (level 1). **c.** Corresponding all-point histogram of the type 1 event. **d.** A representative 5S rRNA type 2 event. The type 2 event starts with random current fluctuations. Then it becomes a single–step blockade (level 1, $\%I_b$=0.578) with many downward burrs. The type 2 events will block the pore until a reversed voltage is applied. **e.** Corresponding all-point histogram of the type 2 event. **f.** A representative 5S rRNA type 3 event. The type 3 event has two blockage levels. The characteristic blockade level measures about 0.775 (level 1). **g.** Corresponding all-point histogram of the type 3 event. **h.** A heat map of t_{off} versus $\%I_b$. $\%I_b$ refers to the blockade amplitude of the level 1 of the three types. The hot map was generated by custom Python codes. **i.** A representative trace containing successive 5S rRNA translocation events. The three types of sensing events of 5S rRNA are clearly recognized from the trace, which are marked with red, blue and green bars respectively. Nanopore measurements were performed as described in Methods. 5S rRNA (Materials, Supplementary Table 1) was added to *cis* with a final concentration of 10 nM.

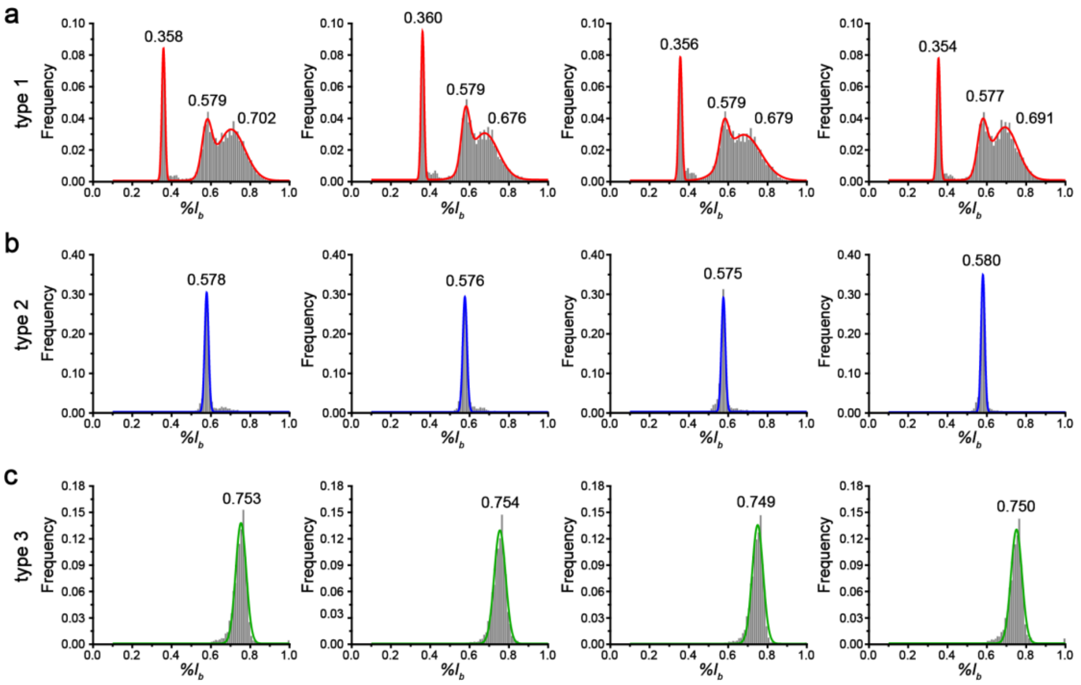

Supplementary Figure 15: The event features of three types of 5S rRNA signals. All-point histograms from representative 5S rRNA type 1 events (**a**), type 2 events (**b**) and type 3 events (**c**) are demonstrated. Distinct patterns were seen in the histograms from different event types. However, the pattern is highly conserved when events of the same type were evaluated. Specifically, all-point histograms of each type 1 event all demonstrate 3 characteristic peaks. Histograms of each type 2 event all demonstrate a single narrower peak with the peak $\%I_b$ at ~ 0.58. Histograms of each type 3 events all demonstrate a single wider peak with the peak $\%I_b$ at ~ 0.75.

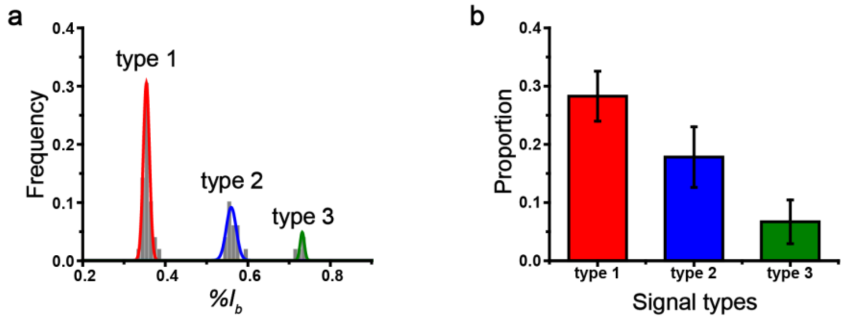

Supplementary Figure 16: Statistics for 5S rRNA sensing events. a. Histogram of $\%I_b$ of the three types sensing events. The distributions of each types follow a Gaussian fitting. **b.** The proportion of the three types sensing events. Type 1 events account for the highest proportion, followed by type 2 and type 3. This suggests that 5S rRNA has a more favored orientation when entering into MspA, which results in the type 1 event. Error bars represent standard deviation, n = 3 independent replicates.

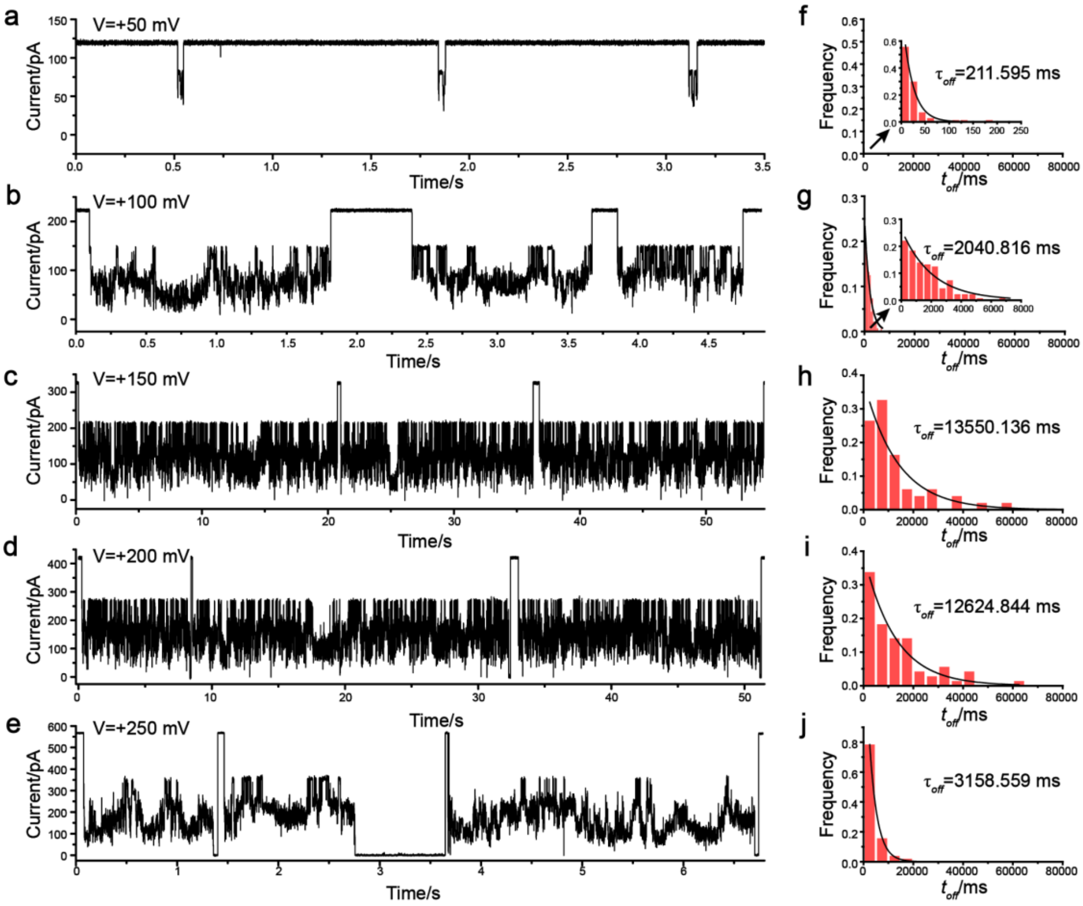

Supplementary Figure 17: Type 1 events of 5S rRNA at different voltages. a-e. Representative type 1 events at +50 mV (**a**), +100 mV (**b**), +150 mV (**c**), +200 mV (**d**) and +250 mV (**e**). **f-j.** Corresponding histograms of t_{off} at +50 mV (**f**), +100 mV (**g**), +150 mV (**h**), +200 mV (**i**) and +250 mV (**j**). τ_{off} increased when the applied potential was increased from +50 mV to +150 mV. However, τ_{off} decreased when the potential was further increased from +150 mV to +250 mV. At +250 mV, all events demonstrate significant fluctuations followed with a deep blockage and a spontaneous restoration to the open pore state. These results indicate that successful translocation of 5S rRNA requires overcoming of a high entropic barrier. A high applied potential would promote translocation of 5S rRNA. The fluctuation noises observed likely result from electrophoretic driven unfolding of its overall structure. Thus, the helix I-down conformation is most likely happening when a type 1 event was observed. The type 1 event is less likely from the loop C or loop E-down conformation since a loop structure is much more difficult to be electrophoretically unfolded than the helix. All measurements were carried out as described in Methods. 5S rRNA was added to *cis* with a final concentration of 10 nM.

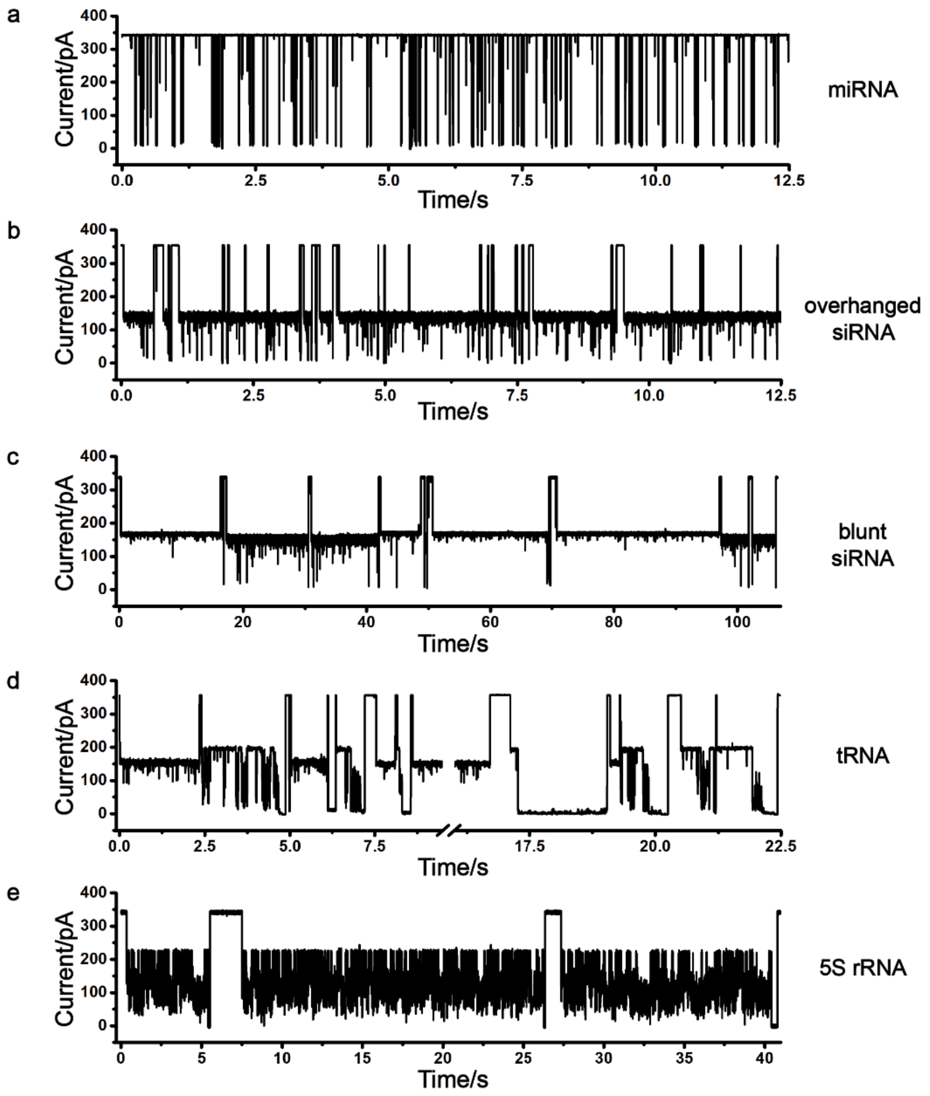

Supplementary Figure 18: Representative traces of RNA translocations. Nanopore measurements were performed as described in Methods. Model analyte of miRNA, siRNA or tRNA was respectively added to *cis* with a final concentration of 200 nM. 5S rRNA was added to *cis* with a final concentration of 10 nM. **a.** A representative trace containing successive miRNA translocation events. Hsa-miR-21 (Supplementary Table 1) is the sole analyte. Translocation of miRNA appears as fast and deep current blockades. **b.** A representative trace containing successive overhanged siRNA translocation events. Hybridized siFoxA1 (Supplementary Table 1) is the sole analyte. Translocation of overhanged siRNA gives rise to characteristic two-step shaped events (Supplementary Fig. 6). **c.** A representative trace containing successive blunt siRNA translocation events. Luciferase siRNA serves as the sole analyte. **d.** A representative trace containing successive tRNA translocation events. Brewer's yeast phenylalanine specific tRNA (Sigma-Aldrich), also termed as $tRNA^{phe}$, serves as the sole analyte. Two types of events with highly distinguishable event characteristics form the majority of all acquired events (Fig. 2b). The current traces from 9.5 s to 16 s has been omitted due to an event with an extremely long residence time. **e.** A representative trace containing successive 5S rRNA translocation events. *E.coli* 5S rRNA recovered from polyacrylamide gels serves as the sole analyte.

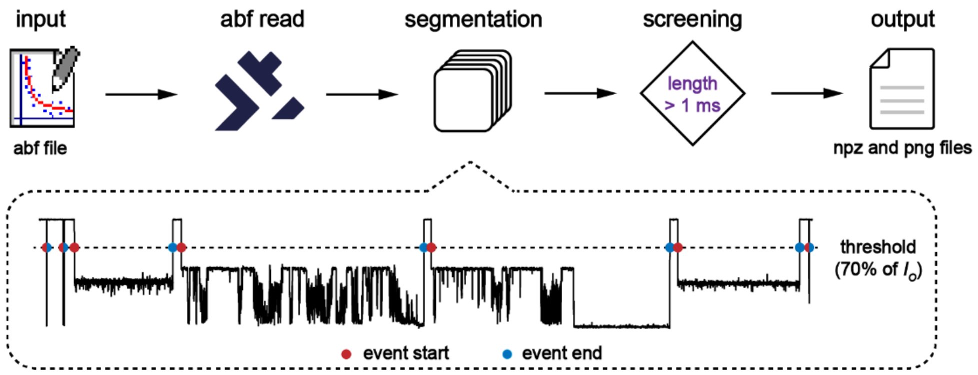

Supplementary Figure 19: A workflow of event extraction. All single channel recordings results were first recorded in .abf files. All .abf files were first imported into the Python environment by the Neo package. Event segmentation was carried out by a threshold search routine custom programmed by Python. Its principle is demonstrated in the dotted box. The threshold of 70% of I_0 was selected because it is much higher than the highest level of all events being studied in this manuscript. No event was missed due to this threshold setting. To perform segmentation, a signal fraction which has a current drop below 70% of I_o followed with a spontaneous restoration to I_o is recognized as an event. To avoid interference from events caused by transient collision of the analyte to the pore, only events with a dwell time more than 1 ms were saved for downstream analysis. The extracted event data were saved in npz format, each accompanied with a png format figure for an ease of visualization.

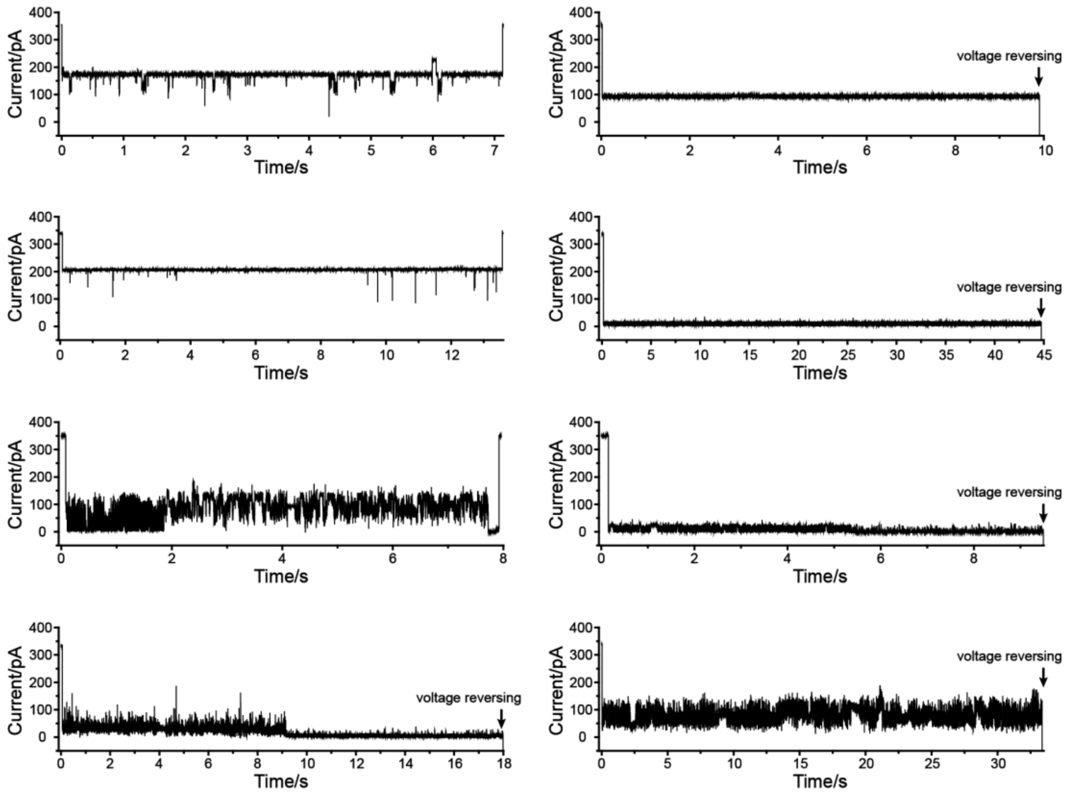

Supplementary Figure 20: Demonstration of "others" events. Nanopore measurements were performed as described in Methods. Events which may result from RNA translocation with an undesired orientation or pore clogging were occasionally observed. Though observable, these events only form a minority of all acquired events and contribute to the type "others" in the machine learning algorithm (Fig. 3a). Please note that a clogged pore can also be manually restored by reversing the applied potential to re-initiate follow-up measurements.

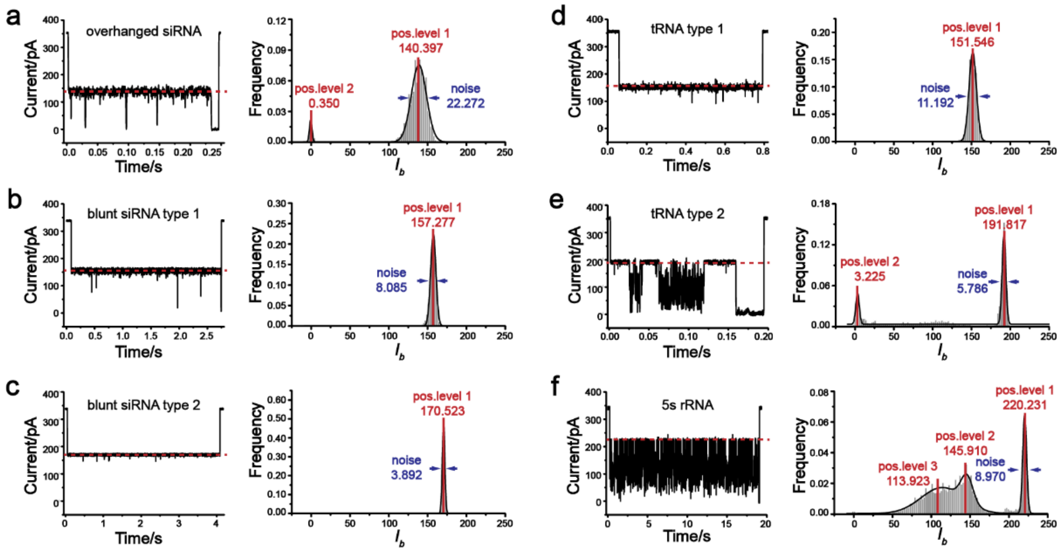

Supplementary Figure 21: Event feature extraction. Multi-peak Gaussian fitting to all point histogram was used to extract event features such as the identity, the position and the noise of the levels from each event. Representative translocation events of overhanged siRNA (siFoxA1) (**a**), blunt siRNA (luciferase siRNA) type 1(**b**), blunt siRNA type 2(**c**), tRNA (tRNA^{phe}) type 1 (**d**), tRNA type 2 (**e**), *E.coli* 5S rRNA (**g**) and their corresponding all point histogram were demonstrated. When there is only one recognizable Gaussian peak in the histogram (**b, c, d**), the identity of the peak is determined as 1. The position and the noise of the peak is determined from the Gaussian fitting results. When more than 2 Gaussian peaks were recognized (**b, a, e, f**), the peak closer to the open pore current was considered to be peak 1 and the other peak is considered to be peak 2, 3. Position and noise of each peak were respectively determined according to the fitting results. With these extracted event features, different types of RNA translocation events are clearly recognizable.

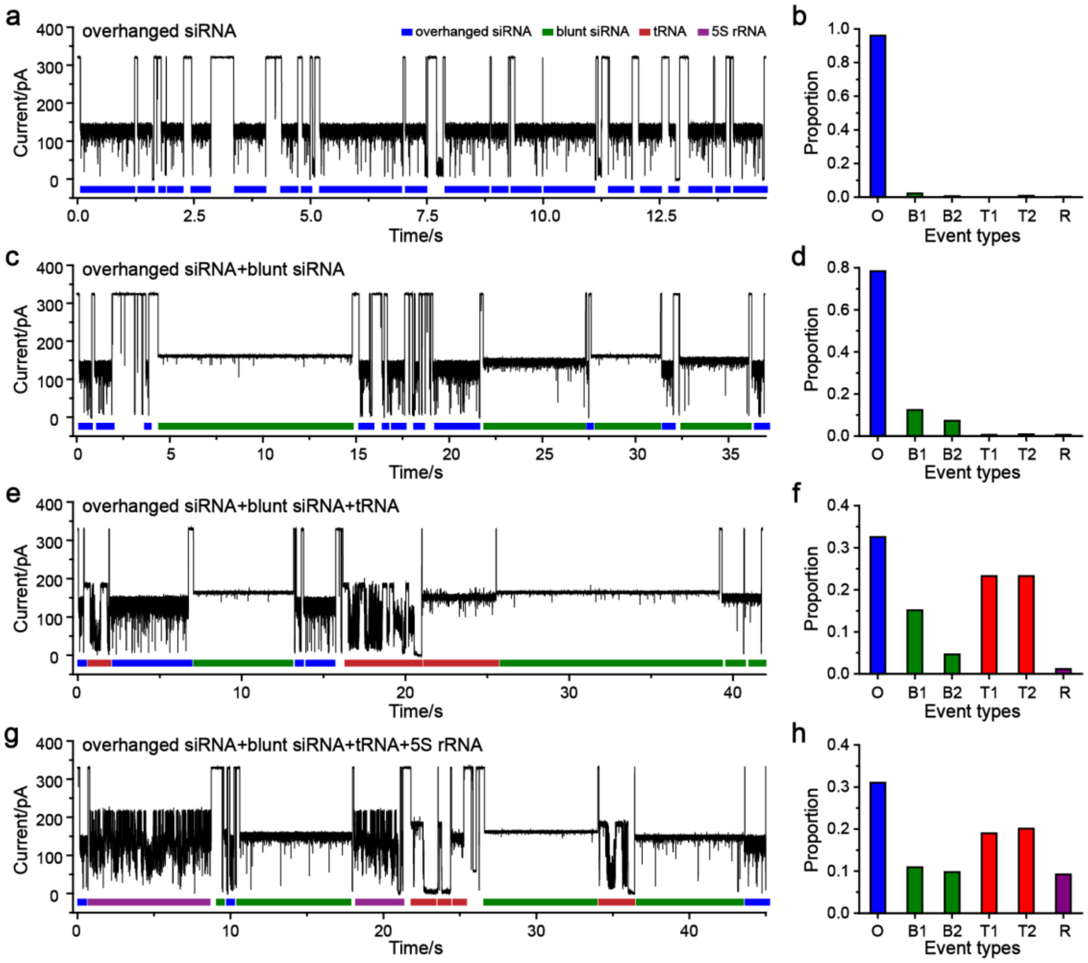

Supplementary Figure 22: RNA type identification with machine learning algorithms. a. A representative trace in the presence of overhanged siRNA (25 nM). Characteristic events of overhanged siRNA (marked with blue bars) were observed in the majority**. b.** Corresponding proportion of different RNA events determined with the Random forest model. 95.8% events were identified as characteristic events of overhanged siRNA. **c**. A representative trace during successive addition of blunt siRNA (10 nM). Except overhanged siRNA events (marked with blue bars), blunt siRNA type 1 and type 2 events (marked with greet bars) were also observed. **d**. Corresponding proportion of different RNA events determined with the Random forest model. Proportions of blunt siRNA type 1 and type 2 events accounted for 0.12 and 0.08. **e**. A representative trace during successive addition of tRNA (450 nM). TRNA type 1 and type 2 events (marked with red bars) appeared in the current trace. **f**. Corresponding proportion of different RNA events determined with the Random forest model. Proportions of tRNA siRNA type 1 and type 2 events accounted for 0.26 and 0.23. **g**. A representative trace during successive addition of 5S rRNA (30 nM). Fingerprint events from the four types of RNA types are clearly recognized from the trace, which are marked with blue, green red or purple bars respectively. **h**. Corresponding proportion of different RNA events determined with the Random forest model. After the addition, the proportion of 5S rRNA events increased from 0 to 0.09. Nanopore measurements were performed as described in Methods. A twenty-minute trace was recorded for each condition.

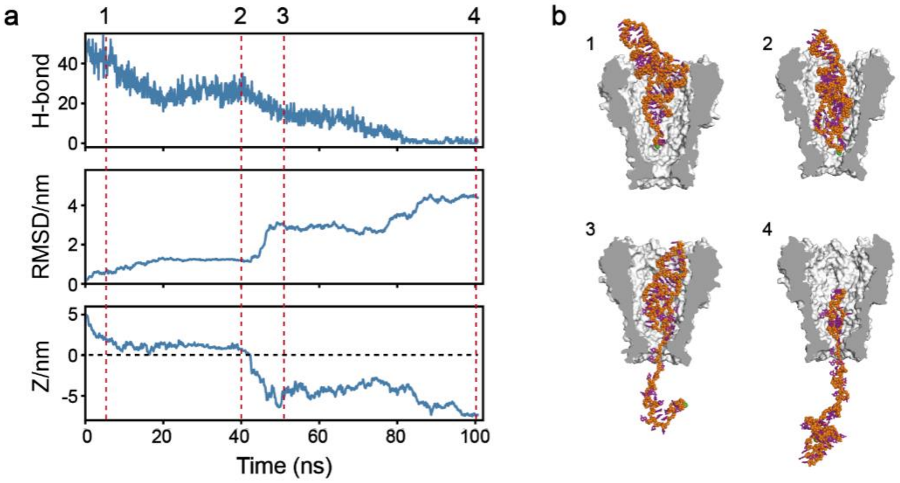

Supplementary Figure 23: Simulated tRNA translocation with the stem-down pose. a. The number of base-pair hydrogen bonds (H-bond), the root mean square deviation (RMSD) from crystal structure and the z-coordinate (Z) of the tRNA as a function of time for a representative MD trajectory with the stem-down pose. An external electric field of 4.0 V/10 nm along the Z-axis was applied to drive the tRNA translocation. **b.** The snapshots of corresponding structures to the four time points labelled in panel **a** (red dashed lines).

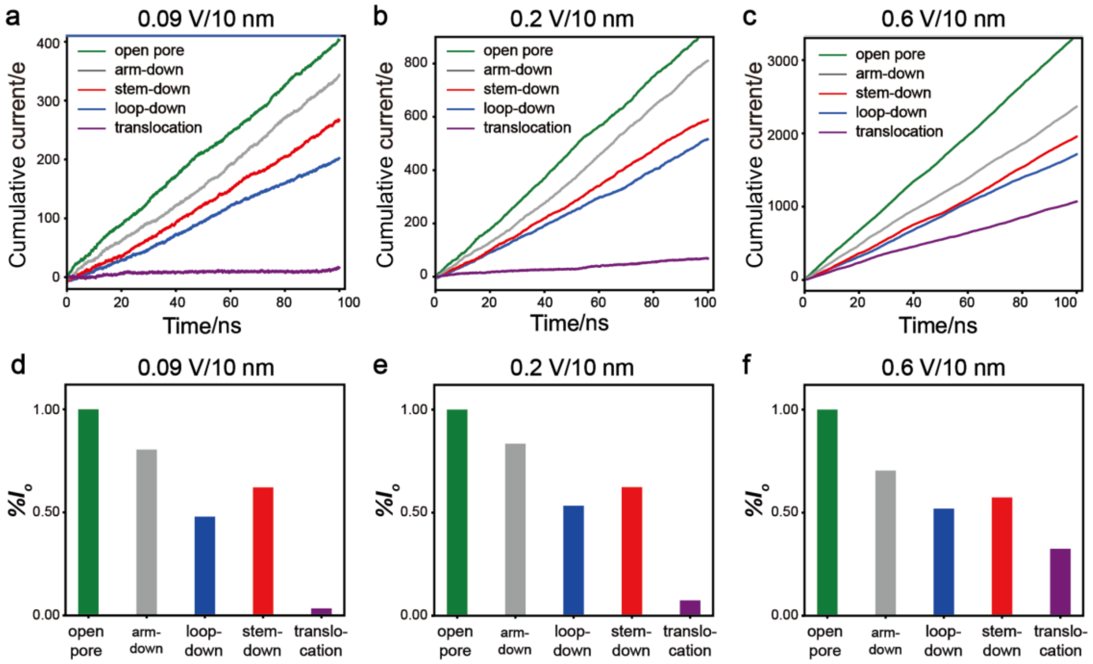

Supplementary Figure 24: Molecular dynamics simulation results. a-c. Cumulative ion currents for the simulations of open pore state (green), the arm-down (gray), the stem-down (red), the loop-down (blue) conformations during the trapping state and when the tRNA was translocating through the pore (purple) under an external electric fields of 0.09 V/10 nm (**a**), 0.2 V/10 nm (**b**) and 0.6 V/10 nm (**c**), respectively. **d-f**. The relative ionic currents for different tRNA orientations under the external electric fields of 0.09 V/10 nm (**d**), 0.2 V/10 nm (**e**) and 0.6 V/10 nm (**f**), respectively.

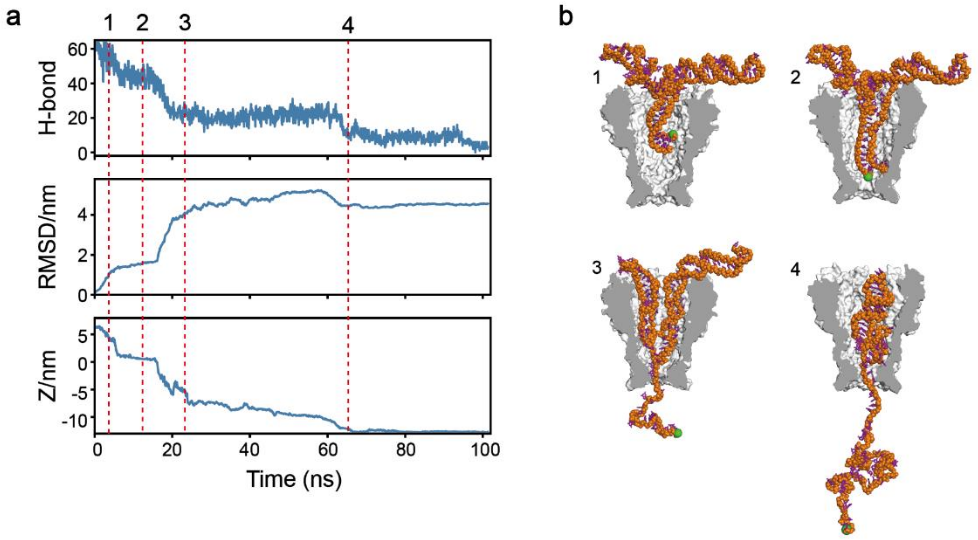

Supplementary Figure 25: Simulated 5S rRNA translocation through MspA. a. The number of base-pair hydrogen bonds (H-bond), the root mean square deviation (RMSD) from crystal structure, and the z-coordinate (Z) of the 5S rRNA as a function of time for a representative MD trajectory with the helix I-down pose. An external electric field of 4.0 V/10 nm along the Z-axis was applied to drive the 5S rRNA translocation. **b.** The snapshots of corresponding structures to the four time points labelled in panel **a** (red dashed lines). Translocation of 5srRNA shows similar behavior as that of tRNA. At the initial stage, the 5srRNA has dramatic deformation without disrupting the base-pair hydrogen bonds as shown by the increase of the RMSD and relatively stable values of the number of formed hydrogen bonds. After reaching the deeper position of MspA, the 5S rRNA gets unfolded by unzipping of the helix domain followed by successful translocation of the leading nucleotide through the pore constriction and further unfolding of the entire structure.

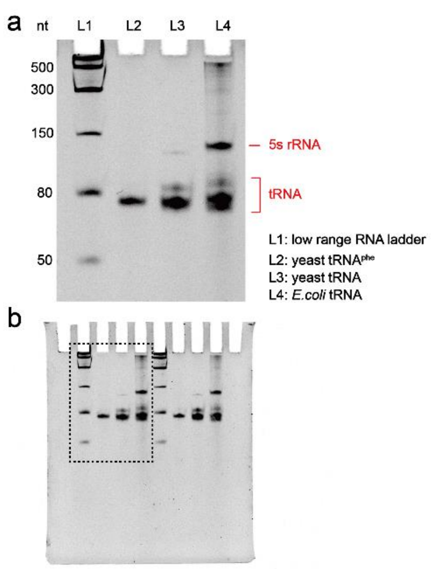

Supplementary Figure 26: Gel electrophoresis of tRNAs from commercial sources. a. 12 % urea-PAGE gel electrophoresis was performed for various tRNA samples from commercial sources, including yeast $tRNA^{phe}$, yeast total tRNA and *E. coli* total tRNA (Sigma-Aldrich). L1: low range RNA ladder; L2: yeast $tRNA^{phe}$; L3: yeast tRNA, L4: *E.coli* tRNA. Gel electrophoresis was continuously run for 60 min with a +180 V applied potential. From the gel results, yeast $tRNA^{phe}$ and yeast tRNA have the desired purity. However, *E.coli* total tRNA contains recognizable contaminations from 5S rRNA (120 nt) and some other RNAs with a higher molecular weight. The identities of these contaminations were determined according to results in the literature[6]. **b.** The uncropped version of the gel in (**a**). The dashed box represents the cropped area.

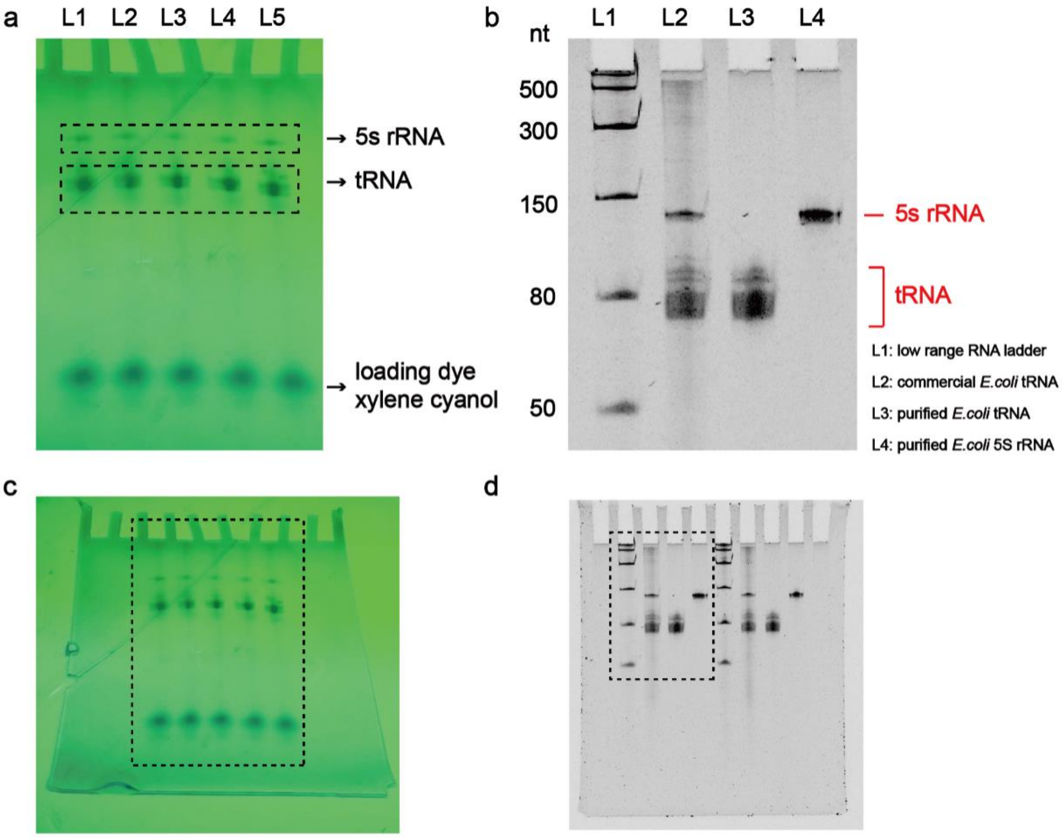

Supplementary Figure 27: Further purification of commercial *E.coli* tRNA. **a.** *E.coli* total tRNA (Sigma-Aldrich) was loaded onto a 12% urea-PAGE gel. Gel electrophoresis was continuously run for 100 min with a +180 mV applied potential. L1-L5: *E.coli* total tRNA. The gel was visualized with a portable UV lamp (254 nm). Three bands were clearly observed and were respectively recognized as 5S rRNA, tRNA and xylene cyanol, according to the published literature[6]. The region marked with dashed boxes was separately excised. The excised gel fragments were treated with the ZR small-RNA™ PAGE Recovery Kit to recover the RNA (Methods). **b.** Recovered RNA fragments characterized using 12% urea-PAGE gel electrophoresis. Gel electrophoresis was continuously run for 100 min with a +180 mV applied potential. L1: low range RNA ladder; L2: commercial *E.coli* total tRNA; L3: excised *E.coli* tRNA; L4: excised *E.coli* 5S rRNA. The recovered *E.coli* 5S rRNA and tRNA were separated from each other and can be separately studied in downstream nanopore measurements. **c.** The uncropped version of the gel in (**a**). The dashed box represents the cropped area. **d**. The uncropped version of the gel in (**b**). The dashed box represents the cropped area.

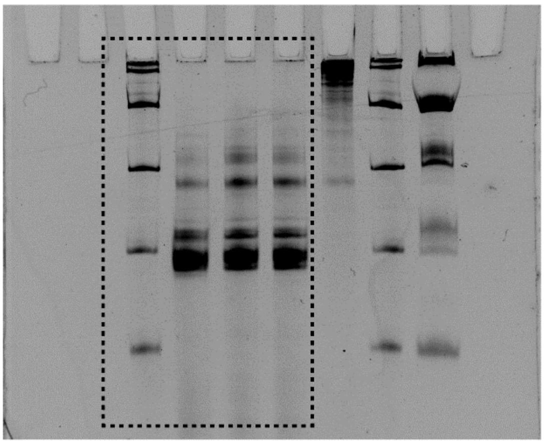

Supplementary Figure 28: The uncropped version of the gel in Fig. 6b. The dashed box represents the cropped area.

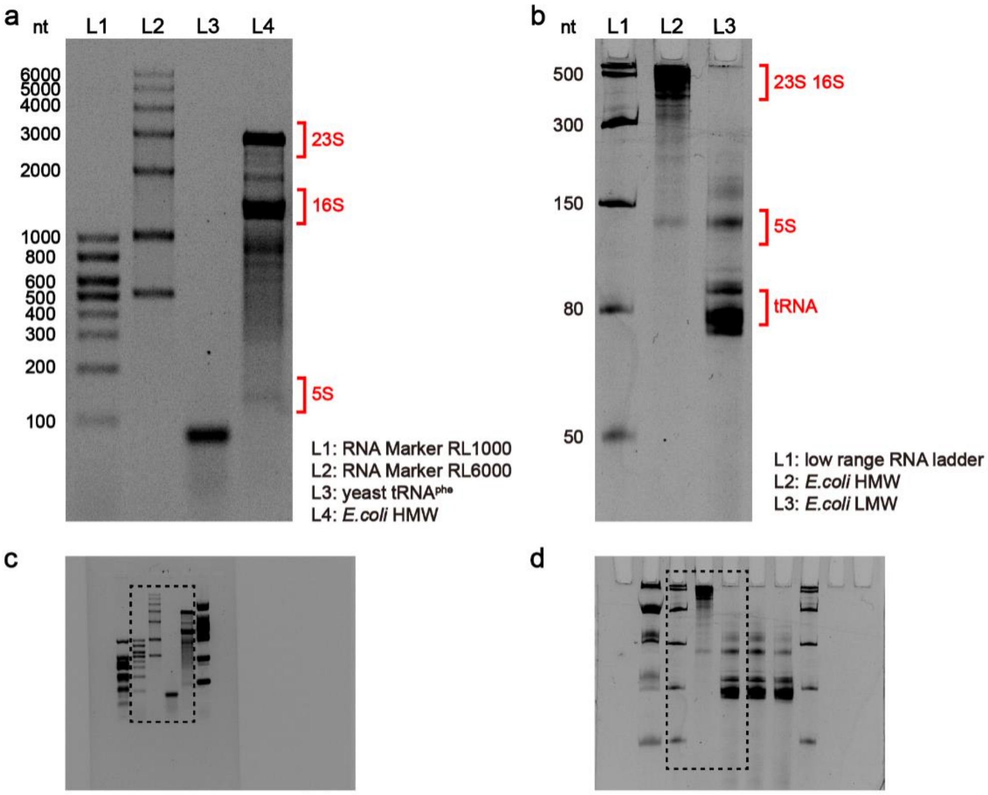

Supplementary Figure 29: Characterization of *E.coli* HMW RNA extraction. *E. coli* HMW RNA was prepared using MiniBEST Universal RNA Extraction Kit (Takara) which specifically extracts RNA with a molecular weight greater than 200 nucleotides[7]. **a.** 1% agarose gel electrophoresis characterization of *E. coli* HMW RNA. Gel electrophoresis was continuously run for 35 min at 4 ^{o}C with a +180 V applied potential. L1: RNA Marker RL1000; L2: RNA Marker RL6000; L3: brewer's yeast tRNA^{phe} from sigma; L4: *E.coli* RNA extraction. From the gel results, the main fractions of the extracted sample are 23S rRNA (2904 nt) and 16S rRNA (1542 nt)[8]. Traces of tRNAs were not observed. **b.** 12 % urea-PAGE gel electrophoresis characterization of *E.coli* HMW RNA. Gel electrophoresis was continuously run for 100 min with a +180 mV applied potential. L1: low range RNA marker; L2: *E. coli* HMW; L3: *E coli* LMW. 12 % urea-PAGE gel electrophoresis was performed to resolve RNAs with a smaller molecular weight[9]. The gel results further confirm that no trace of tRNA were observed from *E.coli* HMW RNA extraction. **c.** The uncropped version of the gel in (**a**). The dashed box represents the cropped area. **d**. The uncropped version of the gel in (**b**). The dashed box represents the cropped area.

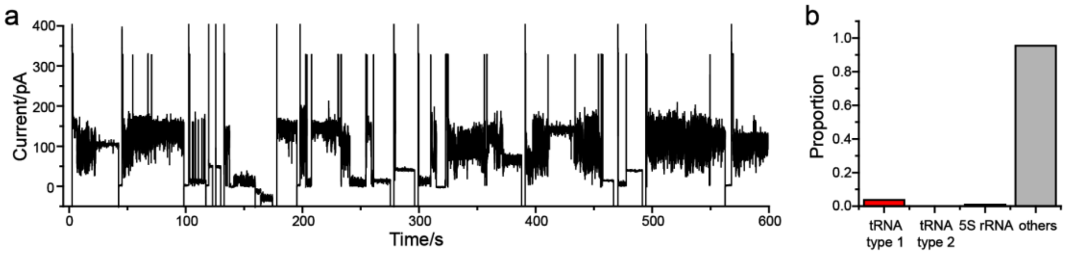

Supplementary Figure 30: *E.coli* HMW RNA sensing. The measurement was performed as described in Methods. *E.coli* HMW RNA was added to the *cis* chamber with a final concentration of 50 ng/µL. **a.** A representative trace of *E.coli* HMW RNA translocation. Most events appear to be extremely long residing. Sometimes HMW RNA may even cause pore clogging. **b.** The proportion of tRNA and 5S rRNA translocation events. No events were recognized as tRNA type 2 events. Only 3.7 % signals were recognized as tRNA type 1 events. The demonstrated results indicate that event characteristics of tRNA type 2 are more reliable in the recognition of tRNA. Events resulted from HMW RNAs can be efficiently excluded by the event characteristics.

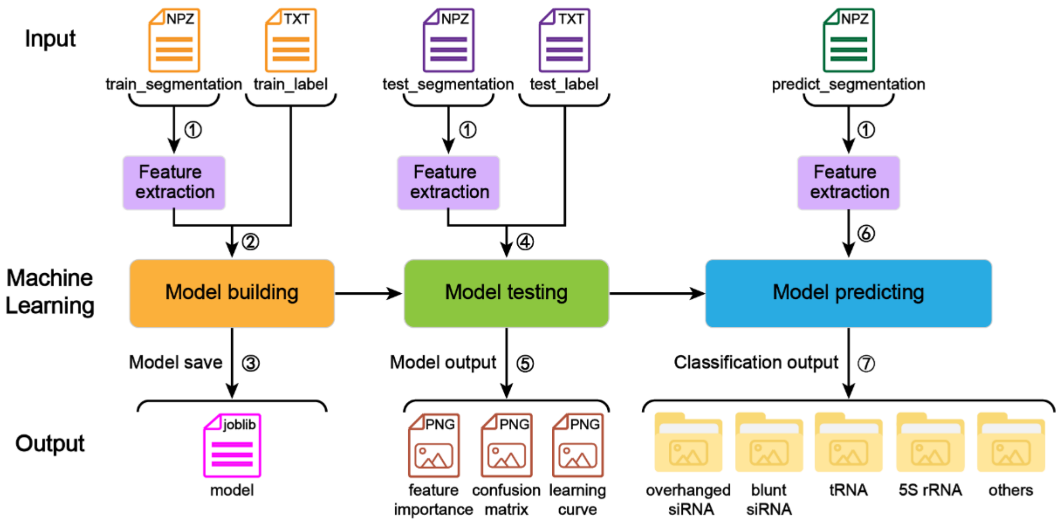

Supplementary Figure 31: The workflow diagram of *RNA-Classification*. The machine learning based algorithm *RNA-Classification,* which was developed and used in this study, has been shared (https://drive.google.com/file/d/17JoqS2JUY-Q0Y4e5Ib0HE4PsexYtEIKq/view?usp=sharing) for validation and further development. Briefly, the inputs contain five files, including the tables of features, labels of the training set and the testing set, as well as the segmentation data of the predicting dataset. Here, the training set and the testing set are model events of different types RNA but with previously known identities. Four sets of data acquired from nanopore measurements with a sequential addition of overhanged siRNA, blunt siRNA, tRNA and 5S rRNA are provided as demo predicting set. The whole workflow is composed of seven steps as below. Step 1: Feature extraction. Eleven parameters of individual events in the segmentation data of dataset are extracted, forming a feature matrix for each event. Step 2: Model building. The feature matrixes and labels of the training set generated by 10-fold cross validation for building the model and fine-tune the parameter. And the best performing trained model is saved into local. Step 3: Model save: The trained model is saved to local for quick loading next time. Step 4: Model testing: The feature matrixes and labels of the testing set are tested by the trained model and validate the performance of the models. Step 5: Model output. Plots of feature importance, confusion matrix and learning curve of the best performing classifiers are generated. Step 6: Model prediction. The feature table of predicting set are loaded to established machine learning model for event identification. Step 7: Classification output. Five folders with sorted events of overhanged siRNA, blunt siRNA, tRNA, 5S rRNA and others are generated.

References

1. Boukhet, M. et al. Probing driving forces in aerolysin and α-hemolysin biological nanopores: electrophoresis versus electroosmosis. *Nanoscale* **8**, 18352-18359 (2016).
2. Piguet, F. et al. Electroosmosis through α-hemolysin that depends on alkali cation type. *J. Phys. Chem. Lett.* **5**, 4362-4367 (2014).
3. Wimmer, E., Maxwell, I.H. & Tener, G.M. A Simple Method for Isolating Highly Purified Yeast Phenylalanine Transfer Ribonucleic Acid. *Biochemistry* **7**, 2623-2628 (1968).
4. Celander, D.W. & Cech, T.R. Visualizing the higher order folding of a catalytic RNA molecule. *Science* **251**, 401-407 (1991).
5. Cate, J.H., Hanna, R.L. & Doudna, J.A. A magnesium ion core at the heart of a ribozyme domain. *Nat. Struct. Biol.* **4**, 553-558 (1997).
6. Farnsworth, R.W., Keating, J., McAuley, M. & Smith, R. Optimization of a Protocol for Escherichia coli RNA Extraction and Visualization. *J. Exp. Microbiol. Immunol.* **5**, 87-94 (2004).
7. Guo, C. et al. Silica nanoparticles induce oxidative stress, inflammation, and endothelial dysfunction in vitro via activation of the MAPK/Nrf2 pathway and nuclear factor-kappa B signaling. *Int. J. Nanomed.* **10**, 1463-1477 (2015).
8. Di Cello, F., Xie, Y., Paul-Satyaseela, M. & Kim, K.S. Approaches bacterial RNA isolation and purification for microarray analysis of Escherichia coli K1 interaction with human brain microvascular endothelial cells. *J. Clin. Microbiol.* **43**, 4197-4199 (2005).
9. Summer, H., Gramer, R. & Droge, P. Denaturing urea polyacrylamide gel electrophoresis (Urea PAGE). *J. Visualized Exp.*, e1485 (2009).
