# Supplementary material for: Structural-profiling of low molecular weight RNAs by nanopore trapping/translocation using Mycobacterium smegmatis porin A
Source: Nat Commun. 2021 Jun 7;12:3368. doi: 10.1038/s41467-021-23764-y (PMC8185011; doi:10.1038/s41467-021-23764-y)
Supplement: Supplementary file 3 — Description of Additional Supplementary Files [file 41467_2021_23764_MOESM3_ESM.docx]

**Description of Additional Supplementary Files**

**Supplementary Movie 1:**

Simultaneous sensing of siRNA, tRNA and 5S rRNA. Electrophysiology measurements were performed as described in Methods. A 1.5 M KCl buffer (1.5 M KCl, 10 mM HEPES, pH 7.0) was placed in cis and a 1 M CaCl2 buffer (1 M CaCl2, 10 mM HEPES, pH 7.0) was placed in trans. Overhanged siRNA (SiFoxA1, 25 nM), blunt siRNA (luciferase siRNA, 10 nM), tRNA (tRNAphe, 400 nM) and 5S rRNA (30 nM) were simultaneously added to cis. Characteristic events of siRNA, tRNA and 5S rRNA were clearly observed from the trace. Assisted by the machine learning algorithm, each event was automatically identified and labelled with letters of O (overhanged siRNA), B1 (blunt siRNA type 1), B1 (blunt siRNA type 2), T1 (tRNA type 1), T2 (tRNA type 2) or R (5S rRNA type 1) respectively.

**Supplementary Movie 2-4:**

Animations of tRNA translocation generated by MD simulation. MD simulations were performed as described in Methods. An external electric field of 4.0 V/10 nm was continuously applied along the direction perpendicular to the membrane plane. The simulations lasted for 100 ns with a time step of 2 fs per frame. In each animation, the tRNA enters the pore with the stem down **(Supplementary Movie 2)**, the loop down **(Supplementary Movie 3)** or the arm down **(Supplementary Movie 4)** conformation.

**Supplementary Movie 5:**

Animation of 5S rRNA translocation generated by MD simulation. MD simulations were performed as described in Methods. An external electric field of 4.0 V/10 nm was continuously applied along the direction perpendicular to the membrane plane. The simulations lasted for 100 ns with a time step of 2 fs per frame. 5S rRNA enters the pore with the helix I down conformation
